# Differentiation of Tracheary Elements in Sugarcane Suspension Cells Involves Changes in Secondary Wall Deposition and Extensive Transcriptional Reprogramming

**DOI:** 10.3389/fpls.2020.617020

**Published:** 2020-12-18

**Authors:** Marcella Siqueira Simões, Sávio Siqueira Ferreira, Adriana Grandis, Jorge Rencoret, Staffan Persson, Eny Iochevet Segal Floh, André Ferraz, José C. del Río, Marcos Silveira Buckeridge, Igor Cesarino

**Affiliations:** ^1^Departamento de Botânica, Instituto de Biociências, Universidade de São Paulo, São Paulo, Brazil; ^2^Instituto de Recursos Naturales y Agrobiología de Sevilla, CSIC, Seville, Spain; ^3^School of Biosciences, University of Melbourne, Melbourne, VIC, Australia; ^4^Department of Plant and Environmental Sciences, University of Copenhagen, Frederiksberg, Denmark; ^5^Copenhagen Plant Science Center, University of Copenhagen, Frederiksberg, Denmark; ^6^Joint International Research Laboratory of Metabolic and Developmental Sciences, State Key Laboratory of Hybrid Rice, School of Life Sciences and Biotechnology, Shanghai Jiao Tong University, Shanghai, China; ^7^Departamento de Biotecnologia, Escola de Engenharia de Lorena, Universidade de São Paulo, Lorena, Brazil; ^8^Synthetic and Systems Biology Center, InovaUSP, São Paulo, Brazil

**Keywords:** C4 grasses, co-expression network, lignin, systems biology, transcriptomics, xylogenic culture, phenylpropanoids

## Abstract

Plant lignocellulosic biomass, mostly composed of polysaccharide-rich secondary cell walls (SCWs), provides fermentable sugars that may be used to produce biofuels and biomaterials. However, the complex chemical composition and physical structure of SCWs hinder efficient processing of plant biomass. Understanding the molecular mechanisms underlying SCW deposition is, thus, essential to optimize bioenergy feedstocks. Here, we establish a xylogenic culture as a model system to study SCW deposition in sugarcane; the first of its kind in a C4 grass species. We used auxin and brassinolide to differentiate sugarcane suspension cells into tracheary elements, which showed metaxylem-like reticulate or pitted SCW patterning. The differentiation led to increased lignin levels, mainly caused by S-lignin units, and a rise in *p*-coumarate, leading to increased *p*-coumarate:ferulate ratios. RNAseq analysis revealed massive transcriptional reprogramming during differentiation, with upregulation of genes associated with cell wall biogenesis and phenylpropanoid metabolism and downregulation of genes related to cell division and primary metabolism. To better understand the differentiation process, we constructed regulatory networks of transcription factors and SCW-related genes based on co-expression analyses. Accordingly, we found multiple regulatory modules that may underpin SCW deposition in sugarcane. Our results provide important insights and resources to identify biotechnological strategies for sugarcane biomass optimization.

## Introduction

Sugarcane (*Saccharum* spp. hybrids), as a C4 plant, is very efficient in generating high yields of biomass with minimal inputs and also has the unique ability within the Poaceae family to accumulate high amounts of sucrose in its mature stem (Diniz et al., 2019). After extraction, sucrose can be directly commercialized as food or fermented to produce the so-called first-generation bioethanol. Sugarcane bagasse, the SCW-rich residue produced after sucrose extraction, is currently burned to generate power and electricity for the production of sugar and ethanol in the mills. However, it can also be used in part as lignocellulosic feedstock, in which SCW polysaccharides are converted into monomeric sugars for fermentation (Klein et al., 2019). The production of lignocellulosic bioethanol from sugarcane bagasse can be achieved by the sugarcane industry, as the feedstock and most of the necessary infrastructure is available (Klein et al., 2019). In a broader perspective, sugarcane bagasse might serve as a renewable and sustainable resource for the production of a plethora of products in biorefineries, including fuels, chemicals and materials. However, due to the complex chemical composition and physical structure of SCWs, processing of plant biomass (including sugarcane bagasse) into downstream products is still considered to be relatively expensive, negatively affecting the transition from an oil-based economy toward a sustainable bio-based economy. Therefore, unraveling the molecular mechanisms underlying SCW deposition in sugarcane is essential for unlocking the economic potential of the bagasse as lignocellulosic feedstock.

The economic potential of sugarcane biomass has stimulated studies aiming to comprehend sugarcane SCW biology, from chemical compositions and physical structure to gene expression and regulation (Bottcher et al., 2013; de Souza et al., 2013; del Río et al., 2015; Costa et al., 2016; Ferreira et al., 2016; Llerena et al., 2019). Despite the recent advances, our understanding of the molecular bases of SCW deposition in sugarcane is still fragmentary, mostly because genetic studies in sugarcane are challenging due to its highly polyploid and complex genome (Cheavegatti-Gianotto et al., 2011). However, recent efforts have provided the scientific community with key sugarcane genomics resources, including the assembly of a 373k gene space of the polyploid genome of the commercial variety SP80-3280 (Souza et al., 2019). In addition, major advances have been achieved in the last few years regarding sugarcane genetic transformation (Lowe et al., 2016; Zhao et al., 2019). Altogether, the availability of such genetic and genomic resources for sugarcane are an excellent and timely basis for a deeper characterization of key molecular aspects of SCW deposition in this important bioenergy crop.

During plant development, SCW deposition occurs in specialized cell types within complex tissues in the plant body, often composed of cells with different morphologies, functions and with intrinsic genetic and developmental programs. The disperse distribution of SCW-depositing cells and the interaction between cell wall components make the study of SCW deposition *in planta* difficult (Kärkönen and Koutaniemi, 2010). In this regard, xylogenic cultures constitute an interesting model system in which a population of relatively homogenous cells growing *in vitro* are induced to differentiate into tracheary elements (TEs; water-conducting xylem cells depositing high amounts of SCW) in response to exogenous stimuli. After induction, changes in cell morphology, cell wall composition and structure, and transcript and metabolite abundances can be investigated by harvesting differentiating cells at different time points (Devillard and Walter, 2014). Xylogenic cultures have been successfully employed to unravel and characterize developmental processes associated with xylogenesis, such as xylem differentiation, SCW deposition and programmed cell death in different plant species (Kubo et al., 2005; Yamagishi et al., 2013; Devillard and Walter, 2014; Ogita et al., 2018). Here, we report on the establishment of a xylogenic culture as a model system to study SCW deposition in sugarcane. Suspension cell cultures were established from friable callus derived from meristematic stem tissues and differentiation of TEs was induced upon treatment with a combination of auxin and brassinolide. We employed biochemical analyses, 2D heteronuclear single-quantum coherence (HSQC) Nuclear Magnetic Resonance (NMR) spectroscopy, and large-scale transcriptomics to investigate the changes in cell wall composition and structure and in transcript abundances associated with the differentiation of TEs and its underlying molecular mechanisms. The establishment and characterization of a xylogenic culture provides new possibilities to understand and manipulate SCW deposition in sugarcane.

## Materials and Methods

### Establishment of Suspension Cell Cultures and Imaging Analyses

Establishment of sugarcane suspension cell cultures was performed as previously described (Cesarino et al., 2013) using transverse sections of meristematic stem tissue from 3-month old of cultivar SP80-3280 as explants. Cultures were maintained in 30 mL liquid MS medium supplemented with 3% (w/v) sucrose and 5 μM 2,4-D, grown in a rotary shaker at 120 rpm in the dark at 25°C and subcultured every 3–4 weeks. Growth rate was measured every 2 days by holding the suspension cells for 10 min in a 15 mL centrifugation tube coupled to a 50 mL Erlenmeyer and measuring the sedimented cell volume. Morphological features of cells were observed using a Zeiss Axio Imager M2 microscope coupled with a Zeiss AxioCam HRc camera. For the induction experiments, cells harvested during the exponential growth phase (i.e., around 10 days after subculture) were first filtered through a 230 μm mesh (CD-1^TM^ 60, Sigma-Aldrich), washed with liquid MS and inoculated (300 mg) in 30 mL liquid MS medium containing 10 μM 2,4-D and 0, 0.2, 1 and 2.5 μM brassinolide. Cultures were kept in a rotary shaker at 120 rpm in the dark at 25°C for 4 weeks. An aliquot of cells was harvested weekly and evaluated for the presence of TEs using a Zeiss Axio Imager M2 microscope coupled with a Zeiss AxioCam HRc camera. Characterization of cell wall thickening patterns was performed by confocal laser scanning microscopy (Zeiss LSM 880 Axio Observer) with excitation at 488 nm.

### Cell Wall Characterization Analyses

Induction of TEs differentiation was performed by transferring cells harvested during the exponential growth phase to liquid MS medium containing 10 μM 2,4-D and 2.5 μM brassinolide. Control cells were inoculated in liquid MS medium containing 10 μM 2,4-D only. After filtration, cells were pooled (six flasks per biological replicate), frozen in liquid nitrogen, lyophilized and ground into powder. Lignin quantification was performed using the acetyl bromide method as previously reported (Fukushima and Kerley, 2011). For non-cellulosic cell wall polysaccharides analyses, cell wall fractionation and monosaccharide profiling were performed as described (de Souza et al., 2013). Hydroxycinnamic acids were released by severe alkaline treatment directly from lyophilized material and determined using HPLC (Masarin et al., 2011).

### 2D-HSQC NMR

Samples from cells after 4 weeks of induction and their corresponding controls were harvested as reported above. Lyophilized samples were successively Soxhlet-extracted with acetone (8 h) and water (3 h) to remove the extractive material and ground in a ball mill. Whole cell-walls were analyzed by 2D-NMR at “gel-state” without previous lignin isolation, according to the method previously published (Kim et al., 2008). 2D-NMR cross-signals were assigned by literature comparison (del Río et al., 2015). A semiquantitative analysis of the volume integrals of the HSQC correlation peaks was performed using Bruker’s Topspin 3.5 processing software. In the aromatic/unsaturated region, the correlation signals of G_2_ and S_2,6_ were used to estimate the content of the respective G- and S-lignin units, the signals for *p*CA_2,6_ and FA_2_ were used to estimate the abundance of the different hydroxycinnamates (as signals S_2,6_ and pCA_2,6_ involve two proton-carbon pairs, their volume integrals were halved). The data were referred to the total content of carbohydrates, estimated from the signals of the anomeric carbons (that mostly correspond to xylose and glucose), and lignin. Data were recorded for two biological replicates, each of them prepared as a pool of six flasks of suspension cells. The cross-signals assigned in the HSQC spectra are listed in Supplementary Table S1 and the relative abundances of the different lignin and hydroxycinnamate units, estimated from the volume integrals of their signals in the spectra, are shown in Supplementary Table S2.

### RT-qPCR Expression Analysis

For the expression analysis, induced and control cells were harvested by vacuum filtration at 0, 12 h, 48 h, and 1 week of treatment, frozen in liquid nitrogen and ground into a fine powder. Total RNA isolation, cDNA synthesis and RT-qPCR analysis were performed as previously described (Simões et al., 2020). Relative expression of each gene was calculated by normalizing it with the geometric mean of the relative quantities of three housekeeping genes, *POLYUBIQUITIN* (*ScPUB*), *UBIQUITIN-CONJUGATING ENZYME 2* (*ScUBE2*), and *GLYCERALDEHYDE 3-PHOSPHATE DEHYDROGENASE* (*ScGAPDH*) (Ferreira et al., 2016). Primers for SCW-related genes were retrieved from previous publications (Casu et al., 2007; Bottcher et al., 2013) or designed within the 3′ UTR region of the gene using Primer3^1^. Primers used in this study are found in Supplementary Table S3. The expression data were analyzed by one-way ANOVA followed by Tukey’s *post hoc* test (*P* < 0.05) along the time series, whereas differences between induced and control samples at each time point were assessed by Student’s *t*-test (*P* < 0.05).

### RNA Extraction, Library Preparation, and Sequencing

The experimental design and cell harvesting for RNAseq analysis were performed as described for the RT-qPCR analysis. Three biological replicates from each time point of control and induced cultures were ground to a powder in liquid N_2_. Total RNA was extracted using ReliaPrep RNA Miniprep System (Promega) followed by DNAse treatment (DNAse RQ1, Promega), according to manufacturer’s instructions. RNA integrity/quality and concentration were assessed using Agilent Bioanalyzer RNA 6000 pico kit in a Bioanalyzer 2100 (Agilent Technologies) and Qubit 2.0 (Thermo Fisher Scientific), respectively. RIN (RNA integrity number) scores of above 7 were used. Poly-A mRNA isolation and cDNA libraries construction were carried out using NEBNext Poly(A) mRNA Magnetic Isolation Module (New England Biolabs) and NEBNext Ultra II Directional RNA Library Prep kit for Illumina (New England Biolabs), respectively, according to manufacturer’s protocols and using unique barcode for each sample. Pooled samples were sequenced in a high-output paired-end run (2 × 150 bp) using an Illumina^®^ NextSeq 500.

### RNAseq Analysis

Low-quality reads (phred score < 20) and adaptor sequences were removed with Trimmomatic (Bolger et al., 2014). Hisat2 v2.1.0 and StringTie v1.3.3 (Pertea et al., 2016) were used for read alignment against the sugarcane reference genome (Souza et al., 2019), read quantification and transcripts assembly, with the following parameters: –min-intronlen 20 –max-intronlen 50000 –dta –rna-strandness RF (for Hisat2); and default parameters for StringTie. Number of reads, filtering and mapping stats are found in Supplementary Table S4. Transcripts annotation was performed using Blast2GO software (Götz et al., 2008) and functional category enrichment analysis was performed with topGO R-package v2.38.1 (Alexa and Rahnenfuhrer, 2020) using biological process category from Gene Ontology (The Gene Ontology Consortium, 2018). Differential expression analysis was performed with DESeq2 (Love et al., 2014) using cutoffs of log2 fold change −1/1 and FDR 0.01. Heatmaps were generated using log2 fold change (FC) of Transcripts Per Million (TPM) normalized by the expression value of each gene at 0 h. Spearman correlation was generated in R using log2 normalized TPM values of all expressed genes and PCA were generated using DESeq2 R-package with the top 5,000 genes with highest expression variance. Venn diagrams were generated with the aid of online tools^2^. Identification of transcription factors (TFs) was carried out using first hit results from blastx searches (BLAST v2.3.0+, cutoff *e*-value 1e-20) against sugarcane and sorghum TFs sequences downloaded from the Grassius TF database^3^. Identification of putative homologs/orthologs in other species was carried out using first hit blastx analysis (cutoff *e*-value 1e-10) against proteomes downloaded from Phytozome. v12.1.6^4^.

### Co-expression Network and Clustering Analysis

Variance stabilizing transformation from DESeq2 was used to estimate gene expression and median absolute deviation (MAD) was used to filter-out non-varying genes. The top ∼30% genes with highest MAD (*n* = 39,744 genes) were used to detect co-expression modules (clusters) using the WGCNA R-package (Langfelder and Horvath, 2008), with the following parameters: power = 14, minModuleSize = 50, deepSplit = 3, maxPOutliers = 0.10, TOMType = “signed,” minCoreKME = 0.7, minKMEtoStay = 0.5, pamRespectsDendro = FALSE, corType = “bicor,” mergeCutHeight = 0.25, networkType = “signed hybrid.” Co-expression networks were visualized using Cytoscape v3.8.0 (Shannon et al., 2003).

## Results and Discussion

### A Combination of Auxin and Brassinolide Induces the Differentiation of TEs in Sugarcane Suspension Cells

To establish a xylogenic culture to study SCW deposition in sugarcane, we generated a suspension cell system from friable callus derived from meristematic stem tissues of greenhouse grown SP80-3280, a cultivar used in Brazilian breeding programs for which a 373k gene space of its genome was recently sequenced and assembled (Souza et al., 2019). Histological analysis using optical microscopy demonstrated that the resulting culture consisted of cells with different morphologies, with both small and generally rounded cells and large and elongated cells, normally aggregated into cell clusters (Supplementary Figure S1). We determined efficient *in vitro* conditions to induce TEs differentiation by transferring suspension cells into various media containing different concentrations of 2,4-D and brassinolide (BL), based on a protocol previously established for lignification of suspension cells of switchgrass (Shen et al., 2013; Rao et al., 2017), a grass species phylogenetically close to sugarcane. Cultures were analyzed weekly for the presence of TEs using optical microscopy during a period of four weeks. Different combinations of such hormones promoted the formation of TEs (Supplementary Figure S2). The combination of 10 μM 2,4-D and 2.5 μM BL was selected because cell growth was maintained, no browning occurred even after 4 weeks of induction and as we observed consistent TEs formation. Interestingly, this combination of phytohormones only induce ectopic lignification in suspension cells of switchgrass, with no changes in cell morphology (Shen et al., 2013; Rao et al., 2017). Brassinosteroids are known to promote xylem differentiation and wood formation (Du et al., 2020) and the addition of these plant steroids to the medium is essential for TEs differentiation in cell cultures of *Arabidopsis thaliana* (Kubo et al., 2005), poplar (Yamagishi et al., 2013), and banana (Negi et al., 2015).

**FIGURE 1:**
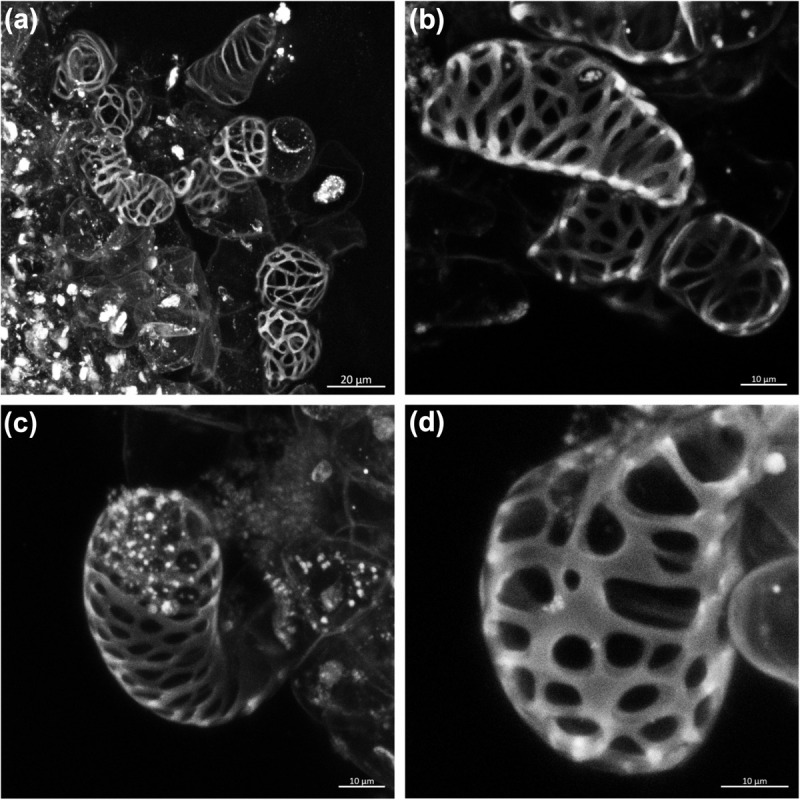
Confocal laser scanning images showing secondary xylem-like tracheary elements in sugarcane suspension cells after 1 week of induction. **(a,b)** Tracheary elements with reticulate patterning of the secondary wall thickening. **(c,d)** Tracheary elements with pitted patterning of the secondary wall thickening.

We used confocal microscopy to characterize SCW deposition in differentiating cells after 1 week of induction. Induced TEs showed thick SCWs deposited in reticulate (Figures 1a,b) or pitted (Figures 1c,d) patterning, both characteristic of later-forming secondary xylem cells (i.e., metaxylem), which has been largely observed in xylogenic cultures of woody species but not those of herbaceous plants (Möller et al., 2006; Yamagishi et al., 2013). We did not observe other types of SCW thickening, such as helical or annular characteristic of early forming xylem cells (i.e., protoxylem). These changes suggest that the phytohormone treatment not only induced cell wall thickening but also induced xylem differentiation in sugarcane suspension cells. To the best of our knowledge, this is the first xylogenic culture developed for a C4 grass species. Finally, an *in vitro* experimental system was recently developed in Arabidopsis, in which vascular development is strongly induced in leaf-disk cultures using bikinin, an inhibitor of glycogen synthase kinase 3 (GSK3) proteins (Kondo et al., 2015). This was due to repression of the inhibitory activity of GSK3s on xylem cell differentiation. We tested the same conditions in sugarcane suspension cells, as well as in leaf disks of sugarcane and sorghum, but found no differentiation of TEs, even though such species harbor homologs of genes encoding GSK3s in their genomes (Supplementary Figure S3). These results suggest that the signaling cascade toward xylem differentiation downstream of GSK3s might not be conserved between eudicots and grasses.

### Differentiation of TEs Is Accompanied by Quantitative and Qualitative Changes in Cell Wall Deposition

**FIGURE 2:**
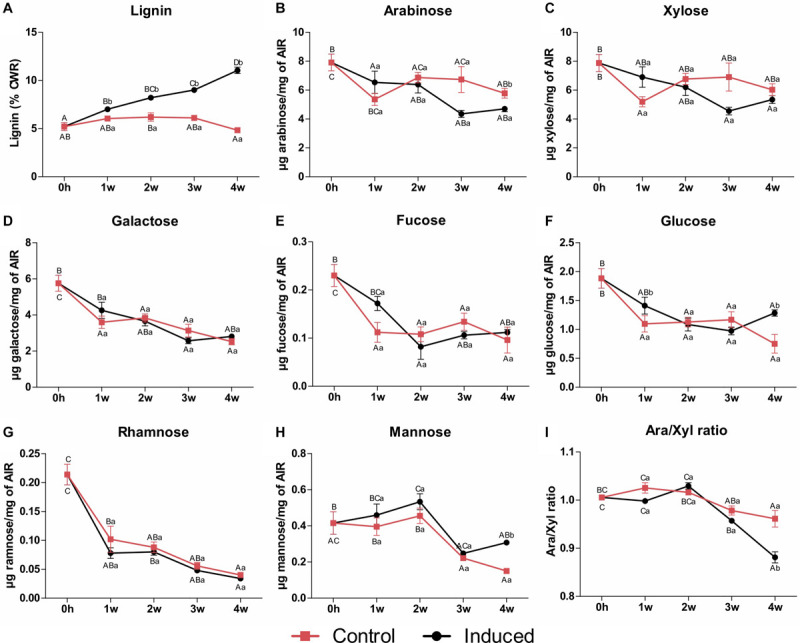
Lignin content and monosaccharide composition of cell wall residues isolated from control and induced sugarcane suspension cells along a time frame of 4 weeks. **(A)** Lignin content determined by the acetyl bromide method. Data are expressed as percentage of CWR. Error bars represent SE (*n* = 4). **(B–H)** Monosaccharide content determined by HPAEC-PAD. Panels indicate **(B)** arabinose, **(C)** xylose, **(D)** galactose, **(E)** fucose, **(F)** glucose, **(G)** rhamnose, and **(H)** mannose. Data are means ± SE (*n* = 5). **(I)** Arabinose to xylose ratio. Data are means ± SE (*n* = 5). Statistical differences were assessed using one-way ANOVA and Tukey’s *post hoc* test (*P* < 0.05) for comparisons along time in the same treatment and using Student’s *t*-test (*P* < 0.05) between control and induced samples at each time point. Different uppercase letters represent significant differences along the time series, whereas different lowercase letters represent significant differences between treatments.

Xylem cells deposit significant amounts of SCWs, and we therefore expected the differentiation of TEs to be accompanied by changes in cell wall content and composition. Hence, lignin content and monosaccharide composition of cell walls from control and induced cells were analyzed as a function of time. Because SCW deposition is a terminal and cumulative process, samples were harvested every week for a period of 4 weeks to allow robust changes to occur. Lignin content, measured as percentage of cell wall residue (CWR), was significantly higher after 1 week of induction, and continued to increase in a roughly linear manner throughout the induction time of 4 weeks reaching up to 12–13% on a CWR basis (Figure 2A). This amount of lignin was equivalent to that observed for maturing internodes, but slightly lower than that of mature internodes of two sugarcane cultivars grown in the field (Bottcher et al., 2013).

Mild hydrolysis of non-cellulosic polysaccharides from alcohol-insoluble residues (AIR) of the sugarcane suspension cells (initial phase, *T* = 0 h) produced mainly xylose and arabinose (Figures 2B–I), suggesting predominance of arabinoxylans. These results are similar to those reported for suspension cell cultures of other grass species, including wheat, rice, switchgrass and even sugarcane (Burke et al., 1974; Rao et al., 2017). High levels of galactose also indicate the occurrence of pectins. By contrast to lignin, the content of most non-cellulosic-related monosaccharides decreased through time in both control and induced cultures (Figures 2B–H). Although the contents of arabinose (Figure 2B) and xylose (Figure 2C) decreased in both cultures when compared to T0h, only the amount of arabinose significantly differed between treatments, being lower in the induced cells after 4 weeks and leading to a significant decrease in the ratio of arabinose to xylose (Figure 2I). Because arabinoxylan is composed of a backbone of β-1,4-linked xylose residues often decorated with arabinose side chains on the O3 position, the arabinose to xylose ratio reflects the degree of arabinoxylan branching/linearity (de Souza et al., 2013; Rao et al., 2017). The significant decrease in arabinose to xylose ratio observed in induced cells indicates that a less branched arabinoxylan is produced during the differentiation process in sugarcane suspension cells. This is in accordance with the results found for internode tissues of different sugarcane hybrids (de Souza et al., 2013; Costa et al., 2016) and species (Llerena et al., 2019) and for stem material of three *Miscanthus sinensis* genotypes (de Souza et al., 2015), suggesting that a less branched polymer is produced in SCW-depositing tissues of grasses. The contents of other non-cellulosic-related monosaccharides showed a consistent and significant decrease through time in both cultures, with little or no differences between the control and the induced cells (Figures 2D–H). These contrasting accumulation patterns between lignin and non-cellulosic polysaccharides were also observed along stem maturation in four experimental sugarcane hybrids (Collucci et al., 2019). These observations suggest that developmental lignification increases cell wall density with a decreased proportion of polysaccharide to lignin ratio (Collucci et al., 2019).

**FIGURE 3:**
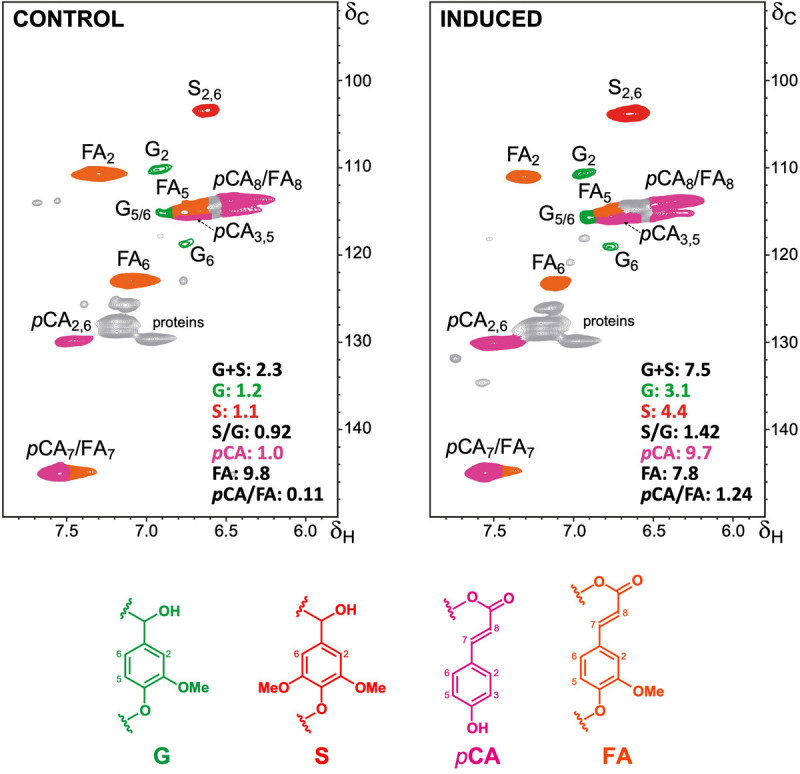
Structural characterization of lignin by NMR. 2D-HSQC-NMR spectra (in DMSO-*d*_6_) is shown for the aromatic/unsaturated regions (δ_C_/δ_H_ 90–150/5.8–8.0) of whole cell walls from control and induced sugarcane cells. Main structures found are: G, guaiacyl lignin units; S, syringyl lignin units; *p*CA, *p*-coumarates; FA, ferulates. The structures are colored to match the assigned contours in the NMR spectra. Integrated values for each monomeric G, S, *p*CA and FA units (as per total lignin and carbohydrate units) and the corresponding S/G and *p*CA/FA ratios are provided.

To further characterize the changes in lignin deposition during the differentiation process, we analyzed cell wall preparations of samples from 4 weeks after induction via 2D HSQC NMR. The main ^1^H/^13^C cross-signals observed in the aromatic/unsaturated region (δ_C_/δ_H_ 90–150/5.8–8.0) of the HSQC spectra corresponded to the aromatic rings and the unsaturated side-chains of the different guaiacyl (G) and syringyl (S) lignin units, and to the wall-bound hydroxycinnamates *p*-coumarate (*p*CA) and ferulate (FA) (Figure 3 and Supplementary Table S1). Signals for *p*-hydroxyphenyl (H) lignin units overlapped with those of proteins and thus were not quantified. Our data revealed higher relative abundance of total lignin units in the induced culture when compared to the control (7.1–7.5 lignin units per 100 lignin + carbohydrate units in induced cells compared to 2.3–2.7 in the control), corroborating the data on lignin quantification. The higher lignin amount in the induced cells was mainly caused by a more pronounced increase in S-lignin units (4.2–4.4 versus 1.1–1.4 in the control) than in the G-lignin units (2.9–3.1 versus 1.2–1.3 in the control), resulting in higher S/G ratios (1.4–1.5 versus 0.9–1.1 in the control) (Figure 3 and Supplementary Table S2). In most xylogenic cultures, G units often represent the main lignin component, most likely because water-conducting cells are typically enriched in G units (Pesquet et al., 2019). Conversely, stress-lignins ectopically deposited in cell cultures of Angiosperms are normally enriched in S units with trace amounts of G units (Shen et al., 2013; Mélida et al., 2015). This is in line with the proposed role of S lignin in plant defense responses against stresses (Cesarino, 2019). The lignin polymer deposited in sugarcane induced cells showed a typical G+S composition with the slightly higher levels of S units than normally found in grass lignins, with a S/G ratio of around 1.2. Nevertheless, similar lignin composition was observed for the bagasse of a hybrid (del Río et al., 2015) and mature internodes of four sugarcane species (Llerena et al., 2019), confirming that what we observe is not due to stress and suggest that our *in vitro* conditions were effective to mimic a natural program controlling xylem differentiation and SCW deposition.

**FIGURE 4:**
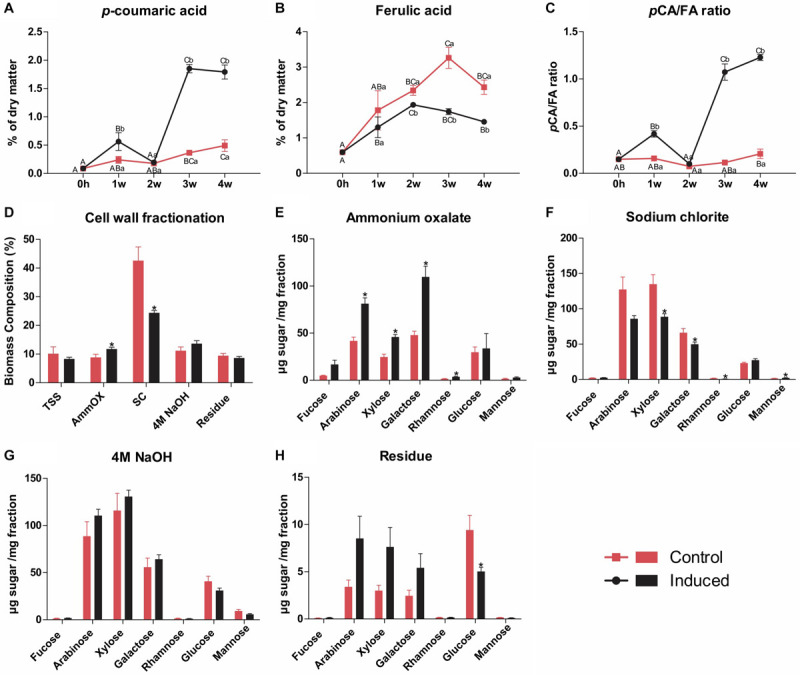
Wall-bound hydroxycinnamates quantification and cell wall fractionation of residues isolated from control and induced sugarcane suspension cells. Hydroxycinnamates were quantified by HPLC after alkaline hydrolysis from samples harvested weekly along a time frame of 4 weeks. Cell wall fractionation was performed in samples from control and induced cells after 4 weeks of treatment. **(A)** *p*-coumarate content determined by HPLC after alkaline hydrolysis. Data are expressed as percentage of dry matter. Error bars represent SE (*n* = 4). **(B)** Ferulate content determined by HPLC after alkaline hydrolysis. Data are expressed as percentage of dry matter. Error bars represent SE (*n* = 4). **(C)** Ratio of *p*-coumarate to ferulate. Data are means ± SE (*n* = 4). **(D)** Yields of total soluble sugars and polysaccharides sequentially extracted upon cell wall fractionation from control and induced cells. Data are expressed as percentage of total biomass ± SE (*n* = 5). **(E)** Monosaccharide composition of the ammonium oxalate extract. Data are means ± SE (*n* = 5). **(F)** Monosaccharide composition of the sodium chlorite extract. Data are means ± SE (*n* = 5). **(G)** Monosaccharide composition of the 4M NaOH extract. Data are means ± SE (*n* = 5). **(H)** Monosaccharide composition of the insoluble wall residue. Data are means ± SE (*n* = 5). For the hydroxycinnamates data, statistical differences were assessed using one-way ANOVA and Tukey’s *post hoc* test (*P* < 0.05) for comparisons along time in the same treatment and using Student’s *t*-test (*P* < 0.05) between control and induced samples at each time point. Different uppercase letters represent significant differences along the time series, whereas different lowercase letters represent significant differences between treatments. For the cell wall fractionation data, statistical differences (indicated by asterisks) were assessed between control and induced samples using Student’s *t*-test (*P* < 0.05).

The hydroxycinnamates *p*CA and FA are chemically bound to different cell wall components in grasses: whereas *p*CA is mainly ester-linked to the γ-OH of S units sidechains in lignin, FA is preferentially ester-linked to arabinosyl residues of arabinoxylan, participating in both polysaccharide-polysaccharide and lignin-polysaccharide cross-coupling reactions (Hatfield et al., 2017). The relative abundance of FA units was slightly lower in the induced culture (7.2–7.8 versus 9.8–10.1 in the control), whereas the relative abundance of *p*CA units was around ninefold higher (9.1–9.7 units versus 1.0–1.3 in the control), leading to higher *p*CA/FA ratios (from around 0.1 in the control to around 1.3 in the induced cells) (Figure 3 and Supplementary Table S2). The higher amounts of *p*CA units in the wall preparations of induced cells are in accordance with its higher lignin levels, as the *p*CA content largely reflects the degree of lignification in grass cell walls (Masarin et al., 2011; Hatfield et al., 2017). These results were further confirmed when the contents of FA and *p*CA were determined in samples for all time points via high-performance liquid chromatography (HPLC) after alkaline hydrolysis. A strong increase in the *p*CA/FA ratio was observed along the time course exclusively in induced cells, mainly caused by a sharp increase in *p*CA content (Figures 4A–C). Altogether, these data corroborate the accumulation of lignin in the induced sugarcane suspension cultures.

To further assess the structural changes in cell wall polysaccharides occurring during the differentiation process, we fractionated AIR preparations of the last time point (i.e., 4 weeks after induction) by sequential extraction with increasingly harsh reagents to release the most weakly to the most tightly bound components of the cell wall (de Souza et al., 2013), which was followed by monosaccharide profiling of the resulting extracts. For the preparation of AIR, the dry matter was extracted with ethanol, which released similar amounts of total soluble sugars (TSS) in both cultures (Figure 4D). Ammonium oxalate, which mainly releases pectins and arabinogalactans loosely bound to the wall by ionic interactions, extracted more material from induced cells when compared to the control (Figure 4D), and monosaccharide profiling showed significantly higher amounts of arabinose, xylose, galactose, and rhamnose in the extracts of the former (Figure 4E). Sodium chlorite, which extracts phenolic-associated wall polysaccharides, released more material from control cells (Figure 4D), whose extract showed higher amounts of xylose, galactose, and rhamnose, but lower amounts of mannose (Figure 4F). No significant differences were observed in the case of 4M NaOH extraction, which typically releases hemicelluloses, tightly bound pectins, arabinogalactans and phenolics, neither for yield (Figure 4D) nor for monosaccharide profile (Figure 4G). Finally, similar yields were found for the insoluble wall residue remaining after all extractions in both cultures (Figure 4D), but higher amounts of glucose were found in control cells in comparison to induced cells (Figure 4H). These results suggest that the differentiation of TEs in sugarcane suspension cells was accompanied by structural changes in cell wall polysaccharides, mainly an increase in the fraction of loosely bound pectins and arabinogalactans and a decrease in phenolic-associated wall polysaccharides, mainly arabinoxylan.

### Differentiation of TEs Results in a Massive Transcriptional Reprogramming in Induced Sugarcane Cells

Because TEs formation is a rather complex process that involves cell differentiation, secondary cell wall deposition and programmed cell death, we expected to observe extensive reprogramming in gene expression. To establish the time frame for the large-scale transcriptomic analysis, the expression of selected SCW-related genes was first evaluated via RT-qPCR in both control and induced cells harvested on 0, 12, 48 h, and 1 week after the beginning of the treatment. This analysis showed an upregulation of lignin biosynthetic genes and *CELLULOSE SYNTHASE* genes already on 12 h after induction, followed by a slight downregulation during later time points (Supplementary Figure S4). In control cells, the expression of these genes remained largely unchanged compared to T0h and were significantly lower than that of induced cells (Supplementary Figure S4). These results confirmed that the selected time frame was effective to unravel expression changes during TEs differentiation. Thus, we chose these time points to perform genome-wide RNAseq analyses.

**FIGURE 5:**
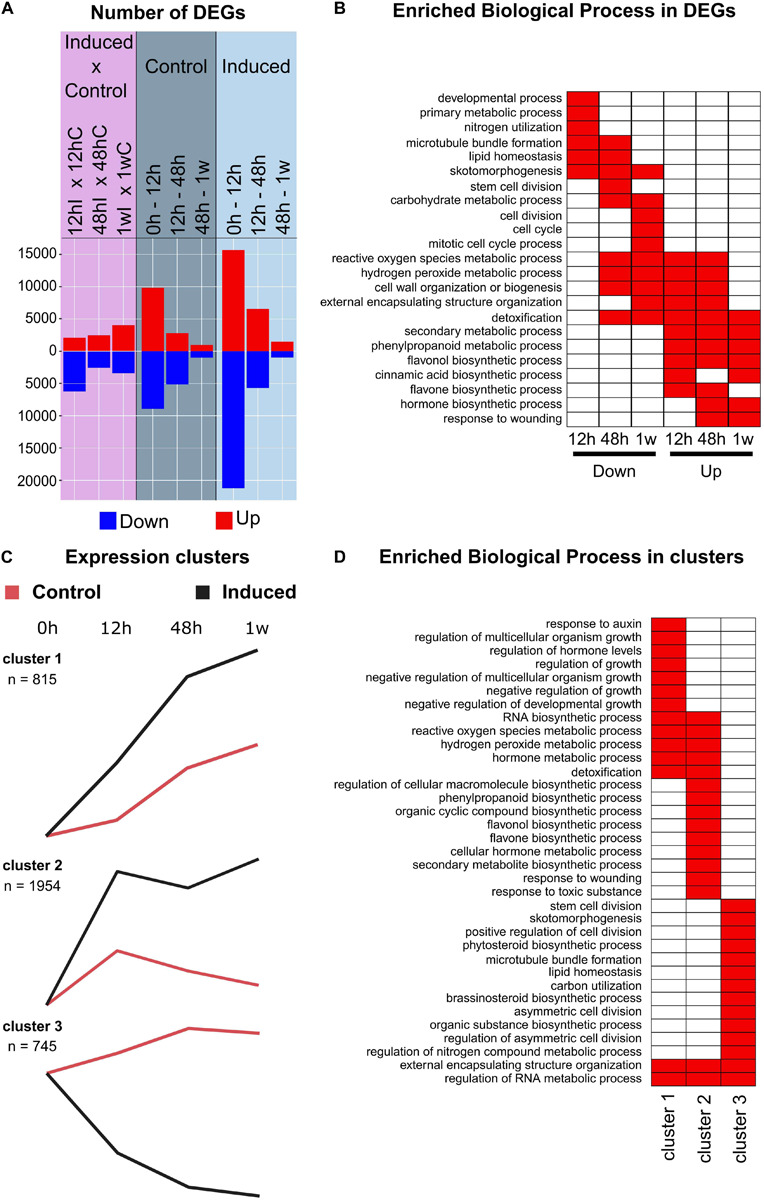
Large-scale transcriptomic analysis of the sugarcane cell cultures. **(A)** Double-sided histograms of the number of differentially expressed genes (DEGs) identified along the time course in control (dark blue shading) and induced (light blue shading) cells, separately, and between control and induced cells in each time point (pink shading). Up- and downregulation is indicated by red and blue color, respectively. **(B)** Enrichment analysis of biological processes terms from Gene Ontology among downregulated and upregulated DEGs found for the comparison control × induced samples for each time point [pink shading in **(A)**]. Enriched biological processes (*P* < 0.0001) in each sample are indicated by red boxes. For brevity, only 23 GO terms are shown. **(C)** Selected clusters/modules whose expression shows distinct patterns between induced and control samples along the time course. Line graphs denote the average of the module eigengene expression, which is the most representative gene expression profile of the module. Average expression of induced and control samples is shown in black and red, respectively. **(D)** Enrichment analysis of biological processes terms from Gene Ontology among genes associated with selected clusters [shown in **(C)**]. Enriched biological processes (*P* < 0.0001) in each cluster are indicated by red boxes. For brevity, only 35 GO terms are shown.

A total of 139,433 genes were detected as expressed in the cells (at least one sample with average TPM ≥ 0.1), approximately one third of all genes annotated in the sugarcane reference genome (Souza et al., 2019). This genome version separates the hom(e)ologous loci in sugarcane, ranging from 1 to 16, which explains the high number of expressed genes observed in the analysis. Spearman correlation and principal component analyses (Supplementary Figure S5) depicted a good correlation among biological replicates and a clear separation between induced and control samples, reflecting the transcriptional changes caused by the differentiation process. Using a threshold of 2x fold change (log2-fold change +1/−1) expression differences and *P* ≤ 0.01 after false discovery rate (FDR) correction, a high number of differentially expressed genes (DEGs) was identified between induced and control cells (Figure 5A and Supplementary Table S5). Compared to the control, the differentiating conditions stimulated a more extensive reprogramming of gene expression, especially in the first time interval (0–12 h transition), but in both cultures the number of DEGs decreased through time (Figure 5A). Venn diagrams were generated for each time interval to identify genes with higher or lower expression exclusively in induced samples (Supplementary Figure S6a). At the 0–12 h time transition (Supplementary Figure S6a), a high number of DEGs were shared between induced and control cells, suggesting that major changes in gene expression occur normally during the progress of the *in vitro* culture. Still, the number of DEGs found exclusively in induced cells was much higher (14,511 down- and 7,226 upregulated) than that found exclusively in the control (2,207 down- and 1,370 upregulated). For latter time transitions, the number of shared DEGs between treatments decreased, as well as the number of DEGs exclusively observed in induced cells. The set of DEGs exclusively found for induced cells provides an interesting resource for gene discovery with respect to vascular development, programmed cell death (PCD) and SCW deposition.

To obtain an overview of the biological processes affected by differentiation of TEs in the sugarcane culture, we undertook enrichment analysis of functional categories from Gene Ontology among DEGs. Biological processes related to ‘secondary metabolic process,’ ‘phenylpropanoid metabolic process,’ ‘flavone biosynthetic process,’ ‘cinnamic acid biosynthetic process,’ and ‘cell wall organization or biogenesis’ were enriched among DEGs upregulated in induced cells when compared to control cells in different time points, whereas ‘cell division,’ ‘mitotic cell cycle,’ ‘microtubule bundle formation,’ and ‘primary metabolism’ were enriched among downregulated DEGs (Figure 5B). When enrichment analyses were performed among DEGs specifically up- or downregulated in induced samples along the time course, similar functional categories were found to be overrepresented (Supplementary Figure S6b). The repression of cell cycle/division and of specific pathways related to primary metabolism concomitantly with the activation of the phenylpropanoid pathway and cell wall biogenesis is consistent with a genetic reprogramming from cell proliferation toward cell differentiation in induced cells, which involves a shift of the cells’ metabolism for the biosynthesis of secondary wall components (Ohtani et al., 2016). A similar shift from primary to secondary metabolism was observed during brassinosteroid-mediated lignification of switchgrass suspension cells (Rao et al., 2017) and during protoxylem formation in etiolated seedlings of dexamethasone (DEX)-inducible VND7 Arabidopsis lines (Li et al., 2016).

A co-expression analysis was further performed to group the genes according to their expression profiles along the time course in both induced and control cultures. Genes with low expression variation among samples were filtered out (see section “Materials and Methods”) and a total of 39,744 genes were used as input. This analysis resulted in 18 clusters of co-expressed genes (Supplementary Figure S7). Among them, three clusters deviated sharply between induced and control samples (Figure 5C). Cluster 1 (*n* = 815 genes) shows genes whose transcript levels linearly and significantly increased along the time course in induced cells when compared to control, whereas transcript levels of genes in cluster 2 (*n* = 1954 genes) showed a sharp increase in the first time interval and remained higher in the following time points. When enrichment analysis was performed for these two clusters individually, cluster 1 was enriched in biological processes related to responses to hormones, negative regulation of growth and cell wall organization and biosynthesis, whereas cluster 2 was largely enriched in functional categories related to secondary metabolism, phenylpropanoids and reactive oxygen species metabolism (Figure 5D). Conversely, cluster 3 (*n* = 745 genes) represents genes for which transcript levels decreased along the time course in induced cells and gradually increased in control cells (Figure 5C), and its enriched categories were mostly related to cell division, microtubule formation and skotomorphogenesis (Figure 5D). Altogether, these results corroborate downregulation of genes involved in growth and proliferation and the upregulation of genes involved in cell specialization and lignification.

To evaluate common and distinctive features in the process of TEs formation between grasses and eudicots, enrichment analysis was applied to public microarray data from an Arabidopsis xylogenic culture induced upon treatment with brassinolide and boron (Kubo et al., 2005) and compared to those found in our study. The comparison with this particular xylogenic culture is convenient because of the similarities regarding system conditions (i.e., *in vitro* cultured cells induced by brassinolide) and time frame (i.e., within 7–10 days of treatment). From the total of 1,705 genes showing more than an eightfold change in expression over the time course, enrichment analyses were performed for three sets of genes with well-defined patterns: (i) down-regulation from *T* = 0 (*n* = 674 genes), (ii) early up-regulation (expression peak between 0 and 4 days; *n* = 341 genes), and (iii) late up-regulation (expression peak between 4 and 10 days; *n* = 512 genes) (Supplementary Table S6). These sets showed analogous expression patterns to clusters 3, 2, and 1 in the sugarcane dataset (Figure 5C), respectively. Although some of the enriched functional categories among down-regulated genes in the Arabidopsis culture were also found for sugarcane cluster 3, the biological processes being repressed along the time course seem to significantly differ between the two systems (Supplementary Figure S8 and Supplementary Table S6). For instance, functional categories related to cell division and microtubule formation enriched among down-regulated genes in the sugarcane culture were not observed for the Arabidopsis set, which was enriched in categories such as “response to stimulus,” “signal transduction” and those involved in housekeeping functions (Supplementary Figure S8 and Supplementary Table S6). When comparing sets of up-regulated genes, perhaps the most striking difference between the two systems was that enrichment with categories related to phenylpropanoid metabolism was observed for early up-regulated genes (cluster 2) in sugarcane but late up-regulated genes in Arabidopsis (Supplementary Figure S8 and Supplementary Table S6). Additionally, the set of late up-regulated genes in the Arabidopsis culture was more strongly enriched with categories related to cell wall deposition and to metabolic pathways related to cell wall components. These data suggest that, despite some similarities, the differentiation of suspension cells into TEs triggered by treatment with brassinolide in sugarcane and Arabidopsis involves largely different transcriptional reprogramming. The fact that bikinin induced the transdifferentiation of mesophyll cells into TEs only in Arabidopsis but not in sugarcane (Supplementary Figure S3) reinforces the differences in this signaling cascade.

### SCW-Related Genes Are Upregulated During the Differentiation of TEs in Sugarcane Suspension Cells

**FIGURE 6:**
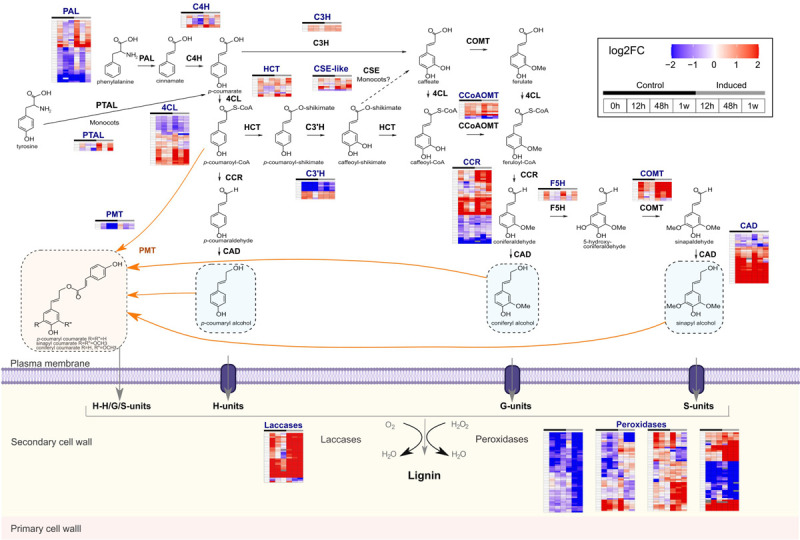
Representation of the monolignol biosynthetic pathway and the secondary cell wall domain embedded with expression data of lignin-related genes. Color code represents expression depicted as log2 fold change (FC) of transcripts per million (TPM) normalized by the expression value of each gene at 0 h. Only genes detected as differentially expressed in at least one comparison are shown. Each heatmap shows one hom(e)ologous genes per line for a given gene family and columns denote the different samples. Black and gray bars on the top of each heatmap indicate control and induced samples, respectively. Figure was adapted from MapMan4 (Schwacke et al., 2019) and includes all enzymatic steps toward monolignol biosynthesis, in addition to monolignol *p*-coumaroylation and oxidation. PAL, phenylalanine ammonia lyase; PTAL, bifunctional phenylalanine/tyrosine ammonia-lyase; C4H, cinnamate 4-hydroxylase; C3H, 4-coumarate 3-hydroxylase; 4CL, 4-coumarate-CoA ligase; HCT, *p*-hydroxycinnamoyl-CoA:quinate/shikimate *p*-hydroxycinnamoyltransferase; C3’H, 4-coumaroyl quinate/shikimate 3-hydroxylase; CSE, caffeoyl shikimate esterase; CCoAOMT, caffeoyl-CoA *O*-methyltransferase; CCR, cinnamoyl-CoA reductase; F5H, ferulate 5-hydroxylase/coniferaldehyde 5- hydroxylase; COMT, caffeate/5-hydroxyconiferaldehyde *O*-methyltransferase; CAD, cinnamyl alcohol dehydrogenase; PMT, *p*-coumaroyl-CoA:monolignol transferase.

Because increased lignin levels were observed for induced cells during the time course and because enrichment analyses showed that genes associated with the phenylpropanoid metabolism were overrepresented among upregulated DEGs in induced samples, a more detailed expression analysis was performed for this metabolic pathway. A total of 227 hom(e)ologous genes involved in the phenylpropanoid and monolignol pathways were detected as differentially expressed in at least one comparison, with varying expression levels and patterns during the time course of the culture (Figure 6 and Supplementary Table S5). Among them, we found putative orthologs of sorghum *BROWN MIDRIB2* (*BMR2*, encoding 4CL; seven genes), *BMR6* (encoding CAD; five genes) and *BMR12* (encoding COMT; seven genes), respectively, which are involved in lignin biosynthesis (Saballos et al., 2008, 2012; Sattler et al., 2009). The identification of individual phenylpropanoid genes that are upregulated in induced cells might be useful to further identify the core set of genes among family members potentially involved in developmental lignification in sugarcane, improving target selection for biotechnological strategies. Previously, large-scale transcriptomics of two sugarcane cultivars contrasting for their lignin content have shown limited success, as only three monolignol biosynthetic genes were differentially expressed between the cultivars (Vicentini et al., 2015). Upregulation of phenylpropanoid/monolignol biosynthetic genes was more pronounced at 12 h and at 1 week of treatment (Figure 6), a similar pattern observed in cluster 2, and 35 from the 227 phenylpropanoid genes were indeed present in this cluster, including putative orthologs of sorghum *BMR2* and *BMR12*. This expression pattern was observed for several genes from the majority of the lignin-related gene families, with exception of *C3’H*, and included *PTAL*, which encodes monocot-specific PALs able to use tyrosine as well as phenylalanine as substrate and shown to provide nearly half of the total lignin deposited in *Brachypodium distachyon* cell walls (Barros et al., 2016). Interestingly, some *CSE-like* genes also showed the same pattern, even though it is still not clear whether CSE is important for lignin biosynthesis in grasses (Barros et al., 2019; Volpi e Silva et al., 2019). Conversely, a different pattern was observed for genes encoding the recently characterized *p*-coumarate 3-hydroxylase, a bifunctional ascorbate peroxidase that catalyzes the 3-hydroxylation of *p*-coumarate to caffeate in the lignin pathway (Barros et al., 2019), as these genes were only slightly upregulated at the transition from 0 to 12 h in induced cells, whereas not differentially expressed in the same time point in the control (Figure 6 and Supplementary Table S7). Consistent with the increased levels of wall-bound *p*CA in induced cells, genes encoding *p*-coumaroyl-CoA:monolignol transferase (PMT), a grass-specific enzyme that acylates monolignols with *p*CA (Petrik et al., 2014), are also upregulated at 12 h in induced samples, but their expression decreased in the following time points.

Tricin is a flavonoid belonging to the flavone subclass that is incorporated into the lignin polymer, and thus it was established as an authentic lignin monomer mainly in the Poaceae family (del Río et al., 2020). In addition to its role in lignification, this flavone also occurs as free tricin, tricin-*O*-glycosides, flavonolignans, and flavonolignan glycosides in sugarcane (Bottcher et al., 2013). The fact that GO term ‘flavonoid metabolism’ was overrepresented among DEGs of induced samples advocated for a more detailed expression analysis of the tricin biosynthetic pathway. Among the genes encoding the flavonoid entry point enzymes chalcone synthase (CHS) and chalcone isomerase (CHI) that were differentially expressed, three *CHS* and seven *CHI* genes were associated with clusters 1 or 2 (Supplementary Table S8), suggesting that they may be responding to the differentiation process. For the genes more downstream in the tricin biosynthetic pathway, ten *CYP93G1* and four *CYP75B4* genes were differentially expressed (Supplementary Table S7), among which three *CYP93G1* and three *CYP75B4* genes belonged to clusters 1 or 2 (Supplementary Table S8). Despite the upregulation of tricin biosynthetic genes, tricin-derived units were not detected in the polymer synthesized by sugarcane induced cells using 2D-HSQC NMR. Although little is known about tricin topochemistry, this flavonoid might be preferentially synthesized and/or incorporated into the lignin of fibers or parenchymatic cells rather than in vessels. Alternatively, tricin glycosylation might preclude its incorporation into the lignin polymer. The major UDP-dependent glycosyltransferases (UGTs) responsible for 7-OH and 5-OH glycosylation of rice flavones (all of them belonging to GT1 family) were recently identified and shown to catalyze the glycosylation of tricin aglycone and tricin-monolignol dimers (Peng et al., 2017). Seven GT1 family members were upregulated in both induced and control cells (Supplementary Figure S7 and Supplementary Table S8), whereas one GT1 was upregulated exclusively in induced cells at 1 week (Supplementary Table S7). The upregulation of *UGT* genes belonging to the GT1 family in induced cells is consistent with the NMR data and suggests that glycosylation might play an important role in modulating tricin incorporation into lignin.

Lignin polymerization occurs in the apoplast and is mediated by laccases and class III peroxidases that are responsible for the oxidation of lignin monomers. These enzymes belong to large multigene families with highly redundant members and diverse functions, making the identification of individual genes involved in lignification a difficult task (Shigeto and Tsutsumi, 2016; Simões et al., 2020). Thus, genes encoding laccases and peroxidases that are specifically upregulated during the differentiation of TEs in the sugarcane culture are strong candidates to be involved in lignin polymerization. A massive number of laccases (31 genes) and peroxidases (197 genes) were differentially expressed in at least one comparison, but with different expression patterns (Figure 6 and Supplementary Table S7). Most laccases were rapidly upregulated after 12 h of induction and their expression levels remained high in the following time points. After 1 week, 27 out of the 31 laccases showed high expression in induced cells when compared to the same time point in control cells (Supplementary Table S7). Expression patterns observed for peroxidases were more diverse, but 78 out of the 197 genes showed higher expression at 12 h and/or 1 week in induced cells compared to control cells. Accordingly, eight laccases and 44 peroxidases shown to be differentially expressed were present in cluster 2 (Supplementary Table S7) and were, therefore, co-expressed with lignin-related genes. Monolignol oxidases are interesting biotechnological targets for lignocellulose optimization because manipulating such terminal players in lignin deposition is less likely to affect plant development due to the loss of other physiologically important phenylpropanoids or to the accumulation of potentially cytotoxic pathway intermediates.

Due to the observed structural changes in cell wall polysaccharides, genes related to cellulose and hemicellulose biosynthesis were also investigated. The cellulose glucan chain is synthesized by a complex of cellulose synthase (CesA) proteins, which are classified as those required for primary (CesA1, CesA3, and CesA8 in rice) and secondary cell wall synthesis (CesA4, CesA7, and CesA9 in rice) (Tanaka et al., 2003; McFarlane et al., 2014). Only four *CesA* genes were upregulated exclusively in induced samples (at the transition from 0 to 12 h), but none of them are potential homologs to rice secondary wall *CesA*s (Supplementary Table S7). Similar results were found for a xylogenic culture of the woody bamboo species *Phyllostachys nigra*, in which SCW-related *CesA*s were not upregulated upon inducing conditions (Ogita et al., 2018). Noteworthy, the determination of CesAs specificities in primary or secondary wall deposition in sugarcane has been problematic. While analyzing the transcripts that are differentially expressed during sugarcane stem development, Casu et al. (2007) observed a major increase in the expression of all *CesA*s (especially those homologous to primary wall *CesA*s in other species) in developing and maturing internodes, tissues in which SCW is actively deposited. This observation led the authors to suggest that in sugarcane there may not be a distinction between primary or secondary wall *CesA* genes or that both types of walls are deposited simultaneously. More recently, tissue-specific transcriptome analysis showed that the expression of putative SCW-related *CesA*s in sugarcane vascular bundles was low (Casu et al., 2015). Altogether, these results suggest that different groups of *CesA* genes might be involved in cell wall deposition in different sugarcane stem tissues or cell types, but currently it is not obvious whether any specificities between PCW and SCW CesAs exist.

The backbone of arabinoxylan, the major hemicellulose found in grass cell walls, is synthesized by the activity of glycosyltransferases (GT) from GT43 and GT47 families, such as the homologs of Arabidopsis IRREGULAR XYLEM9 (IRX9), IRX10, and IRX14 genes (Scheller and Ulvskov, 2010), whereas its side-chain arabinosyl substitutions are incorporated in grasses by xylan arabinosyltransferases (XATs), members of the GT61 family (Anders et al., 2012). In contrast to the reduced number of DEGs encoding *CesA*s, 36 genes from GT43 and GT47 families (putative orthologs of *IRX*s genes) were differentially expressed, 20 of which were upregulated in induced samples, mainly at 12 h (Supplementary Table S7). Moreover, seven *XAT* genes were found upregulated in induced samples, mainly at 12 h (Supplementary Table S7). In addition, two *CslF* and two *CslH* genes, responsible for mixed-linkage glucan biosynthesis (Vega-Sánchez et al., 2012), were also upregulated in exclusively induced samples (Supplementary Table S7). These results are in contrast to those observed for switchgrass suspension cell cultures, in which *CslF* and *GAX*-related genes are mostly downregulated (Rao et al., 2017), most likely because the latter system triggers only ectopic lignification but does not promote cell differentiation, leading to the deposition of different SCWs.

### The Expression of Regulators of SCW Deposition and PCD Is Induced During Differentiation of Tracheary Elements in Sugarcane Suspension Cells

Secondary cell walls deposition is transcriptionally regulated by a hierarchical feed-forward loop of regulators. On the top level, SCW-related NAC TFs, referred to as master switches, directly activate the expression of second-level SCW-related myeloblastosis (MYB) TFs and, together, both activate downstream TFs, mostly MYBs, and the coordinated expression of SCW biosynthetic genes (Ohtani and Demura, 2019). A total of 50 genes encoding NAC TFs were upregulated in induced cells, considering both DEG identification approaches (Supplementary Table S5) and several of them were found in clusters 1 (six genes) and 2 (19 genes), respectively (Supplementary Tables S5, S6), suggesting a potential function during the differentiation process. The sequences of these *NACs* were compared against the Arabidopsis, poplar, rice, maize and switchgrass proteomes using first hit blastx analysis (Supplementary Table S7) to identify their potential orthologs. Two of them (belonging to cluster 1) showed high similarity to SCW-related NACs of different species, named VND4 in Arabidopsis (Zhou et al., 2014), ZmSNW6 in maize (Zhong et al., 2011b) and PvSWN6A/B in switchgrass (Zhong et al., 2015) (Supplementary Table S7). VND TFs, including VND4, directly regulate the expression of a broad range of genes involved in xylem vessel differentiation in Arabidopsis, including SCW biosynthetic and PCD-related genes (Zhou et al., 2014). Additionally, seven NAC TFs that shared high similarities to Arabidopsis *XND1* (three genes) and *ANAC087* (four genes) were associated with cluster 2 (Supplementary Table S7). XND1 was previously characterized as a repressor of SCW deposition and PCD in xylem of Arabidopsis (Zhao et al., 2008). XND1 plays a role in the maturation of metaxylem elements, prolonging the duration of xylem extensibility possibly by counteracting genes such as the SCW NAC master switches VND6/7 and NST1/2 (Zhao et al., 2008). The expression of *XND1* genes in the sugarcane induced cells is thus consistent with the metaxylem-associated patterns of SCW deposition observed for the TEs in the culture. Notably, ANAC087 is a positive regulator of distinct aspects of developmental PCD in Arabidopsis columella root cap cells (Huysmans et al., 2018). The upregulation of sugarcane hom(e)ologs of *ANAC087* during the differentiation process suggests a regulatory role for these TFs in PCD of tracheary elements. Lastly, although not present in any co-expression cluster, two *NAC* genes upregulated from at 12h exclusively in induced samples (Supplementary Table S5) were found to be putative orthologs of Arabidopsis SND2 and poplar PtrNAC154 (Supplementary Table S7), which are both master switches activating SCW deposition (Zhong et al., 2008, 2011a).

MYBs have been extensively characterized as regulators of different branches of the phenylpropanoid pathway. Regarding the SCW transcriptional network, these TFs compose the second level of master switches and most of the third level of direct regulators of SCW biosynthetic genes (Ohtani and Demura, 2019). Ninety-seven genes encoding MYBs were upregulated in induced cells (Supplementary Table S5). Blastx searches showed that several of them (Supplementary Table S7) share high similarity with phenylpropanoid-related MYBs in Arabidopsis, including AtMYB112 (17 genes), a regulator of anthocyanin biosynthesis (Lotkowska et al., 2015), and AtMYB36 (11 genes) that directly and positively regulates the expression of genes involved in the formation of Casparian strips (Kamiya et al., 2015), a root diffusion barrier made of exclusively of lignin (Naseer et al., 2012). In addition, we also found putative orthologs of repressors of phenylpropanoid biosynthesis (Supplementary Table S7), including the Arabidopsis AtMYB4 and AtMYB7 (eight and three genes, respectively) (Wang et al., 2020) and their corresponding orthologs OsMYB108 in rice (Miyamoto et al., 2019), ZmMYB31/42 in maize (Agarwal et al., 2016), and PvMYB4 in switchgrass (Shen et al., 2012). These MYBs are members of subfamily 4 of the R2R3-MYB family that function as transcriptional repressors of monolignol and flavonoid biosynthesis (Agarwal et al., 2016; Wang et al., 2020). Although the upregulation of such transcriptional repressors might be unexpected, similar results were found during SCW formation in seedlings of DEX-inducible VND7 Arabidopsis lines, in which the expression of the repressors *AtMYB7* and *AtMYB32*, another MYB repressor from subfamily 4, increased substantially upon DEX treatment (Li et al., 2016). Moreover, these repressors may act as switches to fine tune the flux through the different branches of the phenylpropanoid pathway in response to environmental and internal signals (Wang et al., 2020). A putative ortholog of the lignin-specific activator AtMYB63 (Zhou et al., 2009) was also upregulated in induced cells after 1 week of treatment (Supplementary Table S5). More importantly, phylogenetic analysis showed that this sugarcane gene may be the true ortholog of *SbMYB60* (Supplementary Figure S9), a co-ortholog of *AtMYB58/63* shown to activate the expression of monolignol biosynthetic genes in *Sorghum bicolor* (Scully et al., 2016). Finally, 33 sugarcane *MYBs* were associated with cluster 1 (three genes) and 2 (30 genes) (Supplementary Table S7), including the putative orthologs of the phenylpropanoid repressors AtMYB4 and OsMYB108 and the anthocyanin activator AtMYB112, reinforcing their involvement in the differentiation process in sugarcane cells. Altogether, these results corroborate previous suggestions that the regulatory network controlling phenylpropanoid genes in grasses employs a dynamic balance of MYB activators and repressors (Agarwal et al., 2016). Alternatively, duplication events followed by sub/neofunctionalization might have contributed to the generation of specialized regulatory activities for some of these TFs, as recently demonstrated for the repressors MYB31/MYB42 in maize, sorghum and rice (Agarwal et al., 2016).

### Co-expression Network Analysis Allowed the Identification of Potential Regulators of SCW Deposition in Sugarcane

**FIGURE 7:**
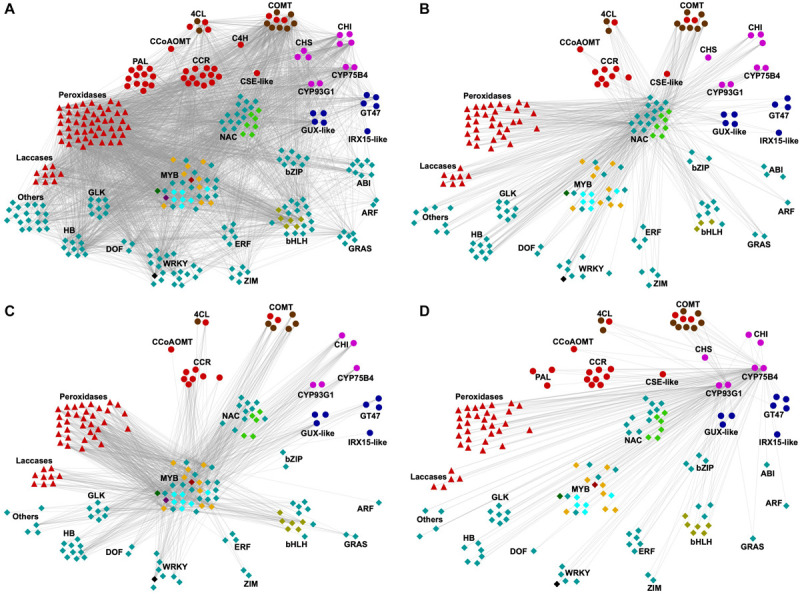
Co-expression network analysis of transcripts associated with cluster 2. Nodes represent genes while edges connect co-expressed genes. For brevity, from all 1954 genes in cluster 2, only those annotated as TF or SCW-related genes and high edge weight are shown (arbitrarily selected as weight > 0.1). Biosynthetic genes are depicted as circles, whereas monolignol oxidases (laccases and peroxidases) and transcription factors are shown as triangles and diamonds, respectively. Genes belonging to the same family were grouped together. Genes were colored as: light red, lignin-related; blue, hemicellulose-related; light purple, flavonoid-related; cyan, transcription factors. Orthologs of sorghum’s *BMR2* (encoding 4CL) and *BMR12* (encoding COMT) genes are denoted as brown circles. TFs of interest are colored differently for convenience: light green, SCW-related NACs (*AtXND1* and *AtANAC087* putative orthologs); light cyan, *AtMYB112* putative orthologs; orange, *AtMYB36* putative orthologs; dark green, *AtMYB4* putative ortholog; dark purple, *OsMYB108* putative ortholog; dark red, *AtMYB63* and putative ortholog; olive, *AtTMO5-like1* putative ortholog; and black, *ScWRKY* among the top 3 edge weight, as mentioned in the main text. **(A)** Network showing all TFs and SCW-related genes (nodes) and connections (edges), filtered by edge weight (>0.1). **(B)** Subnetwork showing only genes directly connected to SCW-related *NACs*. **(C)** Subnetwork showing only genes directly connected to SCW-related *MYBs*. **(D)** Subnetwork showing only genes directly connected to the tricin-specific genes *CYP93G1* and *CYP75B4*.

Co-expression approaches have been employed to identify new genes involved in SCW deposition, based on the observation that genes with similar function are often transcriptionally coordinated (Ruprecht et al., 2017). To identify regulators of SCW deposition in sugarcane, co-expression network analysis was applied to the transcriptomics dataset using weighted correlation network analysis (WGCNA) and the sugarcane homologs to sorghum *BMR2* and *BMR12* as baits. These genes were chosen because their function in lignification is well established in sorghum, a phylogenetically close species to sugarcane. In addition, both genes were associated with cluster 2, which was the cluster enriched with GO terms related to secondary and phenylpropanoid metabolisms (Figure 5D). The method used for network analysis, WGCNA, provides weight to connections between genes, allowing the identification of strongly co-expressed genes (high edge weight). The resulting network comprised 1954 genes (nodes), from which three functional categories are highlighted in Figure 7A: SCW biosynthetic genes (63 genes), monolignol oxidases (59 genes), and TFs (175 genes). Among biosynthetic genes, members of all three families involved in the general phenylpropanoid pathway were present in the network, in addition to families of some of the downstream steps of the lignin-specific (*CCR*, *CCoAOMT*, *CSE-like*, and *COMT*) and the flavonoid/tricin-specific branches. Genes related to hemicellulose biosynthesis, such as *GUX-like*, *IRX15-like*, and *GT47*, and several monolignol oxidases were also part of this network. These co-expressed genes may provide important functions to SCW deposition in sugarcane and are potential targets for biotechnological strategies. The sugarcane co-expression network enriched in SCW-related genes comprised a similar set of TF families to those obtained in studies on SCW deposition in *Miscanthus* spp., Arabidopsis and switchgrass (Hu et al., 2017; Rao et al., 2019), consistent with the evolutionary conservation of the core regulatory network for SCW deposition (Ohtani and Demura, 2019). In addition to *NACs* and *MYBs* (21 and 34 genes, respectively), a total of 120 genes encoding TFs from different families were also co-expressed with the target lignin biosynthetic genes, including WRKY, bHLH, DOF, bZIP, ARF, GLK, GRAS, ZIM, ABI, ERF, and HB (Figure 7A and Supplementary Tables S8, S9). Several members of the NAC, MYB, WRKY, ERF, and ZIM families have been previously characterized as SCW or lignin biosynthesis regulators (Ambavaram et al., 2011; Vélez-Bermúdez et al., 2015; Rao et al., 2019) whereas members of the MYB and bHLH families are known regulators of flavonoid biosynthesis (Xu et al., 2015). For instance, several *MYBs* in the network are putative orthologs of the lignin-specific activators *AtMYB63* (Figure 7A, dark red diamond within the *MYB* neighborhood; Supplementary Table S9), the anthocyanin activator *AtMYB112* (Figure 7A, light cyan diamonds; Supplementary Table S9), Casparian strip regulator *AtMYB36* (Figure 7A, orange diamonds; Supplementary Table S9) and the phenylpropanoid repressors *AtMYB4* and *OsMYB108* (Figure 7A, dark green and dark purple diamonds, respectively; Supplementary Table S9).

To gain further insights into the potential role of the co-expressed *NACs* and *MYBs* as regulators of SCW deposition, subnetworks were produced by selecting only genes directly connected to either *NACs* (Figure 7B and Supplementary Table S10) or *MYBs* (Figure 7C and Supplementary Table S10) and also showing stronger connections (edge weight > 0.10). The subnetwork of genes co-expressed with *NACs* comprised 185 nodes and several of them were SCW-related genes including phenylpropanoid/monolignol (22 genes), tricin (8 genes) and hemicellulose biosynthetic genes (8 genes), peroxidases (33 genes), laccases (8 genes) and TFs from the same gene families as observed for the initial network (93 genes), with the exception of the ABI family (Figure 7B and Supplementary Table S10). Among these TFs, 23 were from the MYB family, including putative orthologs of *AtMYB4*, *AtMYB36* and *AtMYB112* (Figure 7B and Supplementary Tables S9, S10). Similarly, MYB subnetwork (Figure 7C and Supplementary Table S10, 175 genes) also comprised genes related to phenylpropanoid/monolignol (17 genes), tricin (5 genes) and hemicellulose biosynthesis (7 genes), peroxidases (33 genes), laccases (9 genes), and TFs (70 genes). Moreover, 11 out of the 15 co-expressed *NACs* in this subnetwork were homologous to SCW-related *NACs* of other plant species, including the putative orthologs of Arabidopsis *XND1* (three genes) and *ANAC87* (two genes) (Figure 7C and Supplementary Tables S9, S10). The observation that several *NACs* and *MYBs* are strongly connected to lignin, tricin and hemicellulose biosynthetic genes in the network indicates that these TFs might regulate the biosynthesis of SCW components in sugarcane.

As the NAC-MYB-based gene regulatory network has been demonstrated to be highly evolutionary conserved across the plant kingdom, research focus is now shifting toward the identification of novel molecular hubs connecting developmental/environmental signals in SCW deposition (Ohtani and Demura, 2019). Recent studies have characterized developmental upstream regulators of the NAC-MYB network in both eudicots and grasses, among which TFs belonging to the WRKY family. WRKY12, which belongs to group IIc of the WRKY family, is a putative regulator of pith cell maintenance by repressing SCW deposition in pith cell walls in Arabidopsis and *Medicago truncatula* (Wang et al., 2010). The function of WRKY12 as repressor of SCW accumulation has been further confirmed for its orthologs in switchgrass and maize (Gallego-Giraldo et al., 2016). WRKY15, a member of the group IId of the WRKY family, was identified as a negative regulator of TEs differentiation by suppressing the expression of *VND7* in procambial cells of Arabidopsis roots (Ge et al., 2020). Interestingly, although not the closest homologue to *AtWRKY12* or *AtWRKY15*, a *ScWRKY* gene (Figure 7, black diamond; Supplementary Table S9) was among the top three highly co-expressed genes (higher edge weight) in both *NACs* and *MYBs* subnetworks (Figures 7B,C and Supplementary Table S10), suggesting a role for this TF in regulating SCW-related processes in sugarcane. The identification of WRKYs from different groups within the family with a role in regulating the NAC-MYB network in different cell types indicates that there is still much to be explored in terms of regulation of SCW deposition. For instance, TFs from the GRAS family are interesting candidates for further analysis, as these TFs have been systematically found as co-expressed with SCW biosynthetic genes in different species, such as Arabidopsis (Rao et al., 2019), bamboo (Ogita et al., 2018), switchgrass (Rao et al., 2017, 2019), and Miscanthus (Hu et al., 2017), and were also found in the sugarcane network (Figure 7A).

The recent recognition of tricin as an authentic lignin monomer in grasses brings the question on whether tricin biosynthesis is controlled by the same gene regulatory network employed for the biosynthesis of the canonical monolignols. Sugarcane tricin biosynthetic genes were co-expressed not only with lignin biosynthetic genes (Figure 7A) but were also directly connected with *NACs* and *MYBs* in their corresponding subnetworks (Figures 7B,C and Supplementary Table S10). This observation suggests that tricin and lignin biosynthetic genes share some common regulatory mechanisms. To gain further insights into the regulation of tricin biosynthesis, we produced a subnetwork by selecting only genes directly connected to the tricin biosynthetic genes *CYP93G1* and *CYP75B4* (Figure 7D and Supplementary Table S10), which was followed by the identification of the 83 co-expressed TFs. Among them, the TF family with the highest number of members in this subnetwork was MYB with 20 genes (including the putative orthologs of *AtMYB4*, *AtMYB36*, *AtMYB63*, and *AtMYB112*), followed by NAC with 16 genes (including the homologs of *XND1* and *ANAC087*) (Figure 7D and Supplementary Table S10). Interestingly, the putative ortholog of *OsMYB108* was not present in this subnetwork. Although not comprehensively characterized, *OsMYB108* loss-of-function not only resulted in higher lignin levels but also in the augmentation of *p*-coumaroylated and tricin lignin units in rice cell walls (Miyamoto et al., 2019). The fact that the ortholog of *OsMYB108* was present in cluster 2 and, thus, is co-expressed with tricin biosynthetic genes, but was not directly connected with such genes in the tricin subnetwork suggests a potential role in regulating tricin biosynthesis, although this regulation might not be direct. Additionally, R2R3 MYBs are known to physically interact with bHLHs and WD-repeat proteins to form MBW ternary complexes and, thus, regulate flavonoid biosynthesis (Xu et al., 2015). bHLH was the third family with the highest number of members in the tricin subnetwork (nine genes); however, none of the sugarcane *bHLH* in this subnetwork or the complete network are putative orthologs of *bHLHs* known to regulate the phenylpropanoid metabolism (Supplementary Table S9). Nevertheless, six sugarcane *bHLHs* present in the subnetwork were putative orthologs of Arabidopsis *TMO5-like1* (Figure 7D and Supplementary Table S9), which has been shown to induce procambium cell proliferation and the establishment of xylem precursor cells (Ohashi-Ito et al., 2014). Further characterization of these sugarcane *bHLHs*, along with other TFs co-expressed with tricin and lignin biosynthetic genes, might help understanding some of the molecular mechanisms underlying grass-specific aspects of vascular development, flavonoid biosynthesis and SCW deposition.

## Conclusion

Here, a combination of auxin and brassinosteroids triggered the differentiation of TEs in sugarcane suspension cells, which results in massive changes in cell wall structure and global gene expression profiles. This xylogenic culture constitutes a first such system in C4 grasses and presents an interesting model system to study different aspects of vascular development and SCW deposition. Our data provide insights into structural features of sugarcane SCWs and, most importantly, may enable identification of key genes involved in the regulation, biosynthesis and polymerization of the different components of SCW. Accordingly, several genes encoding enzymes involved in the biosynthesis of SCW components, monolignol oxidases and TFs were identified in co-expression analyses and are excellent candidates for future functional analyses. Because the sugarcane xylogenic culture consists in an *in vitro* system, it may not completely represent the natural process of xylem formation occurring *in planta*, and future functional validation of these candidate genes is still necessary. Ultimately, these findings provide a basis for the genetic engineering of sugarcane to optimize its biomass for the production of downstream bioproducts in biorefineries.

## Data Availability Statement

The datasets presented in this study can be found at Sequence Read Archive repository (SRA – NCBI), https://www.ncbi.nlm.nih.gov/sra, under the BioProject accession number PRJNA658777.

## Author Contributions

IC, MS, and SF conceived the study. MS established the cell cultures and the differentiation protocol and performed most of the experiments. EF supervised the establishment of the sugarcane cell cultures. AG and MB supervised the cell wall characterization and fractionation analyses. JR and JCR performed and analyzed the NMR data. SF performed and analyzed the RNAseq and co-expression data. AF supervised the determination of wall-bound hydroxycinnamates. SP contributed to experimental design and data analyses. IC and SF wrote the manuscript with further input of all co-authors. All the authors contributed to the article and approved the submitted version.

## Conflict of Interest

The authors declare that the research was conducted in the absence of any commercial or financial relationships that could be construed as a potential conflict of interest.

## Footnotes

1 http://bioinfo.ut.ee/primer3-0.4.0/

2 http://bioinformatics.psb.ugent.be/webtools/Venn/

3 http://grassius.org

4 https://phytozome.jgi.doe.gov/
