# Supplementary figures and images for: Differentiation of Tracheary Elements in Sugarcane Suspension Cells Involves Changes in Secondary Wall Deposition and Extensive Transcriptional Reprogramming
Source: Front Plant Sci. 2020 Dec 18;11:617020. doi: 10.3389/fpls.2020.617020 (PMC7814504; doi:10.3389/fpls.2020.617020)

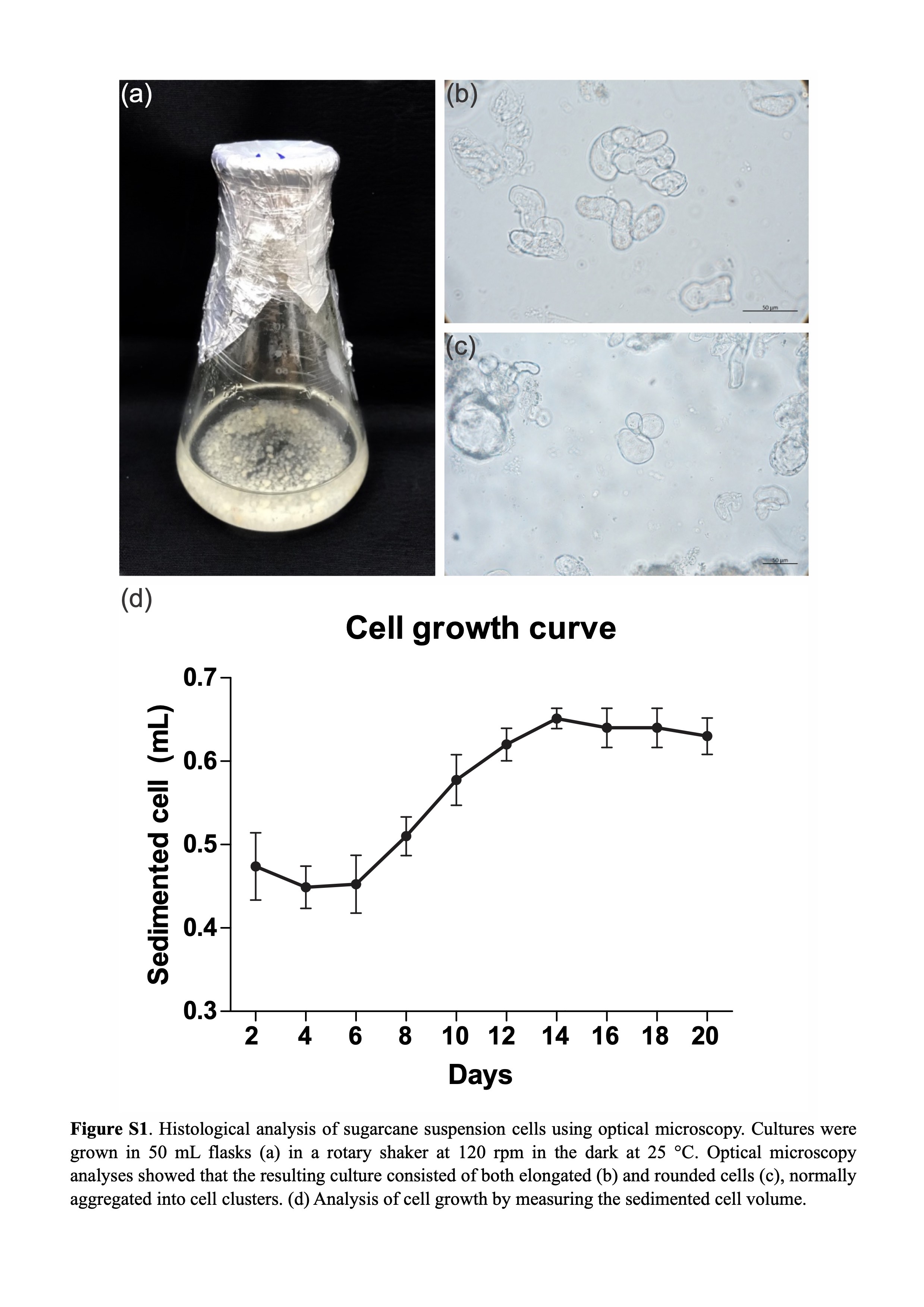

Supplement: Supplementary file 1 [file Image_1.JPEG]

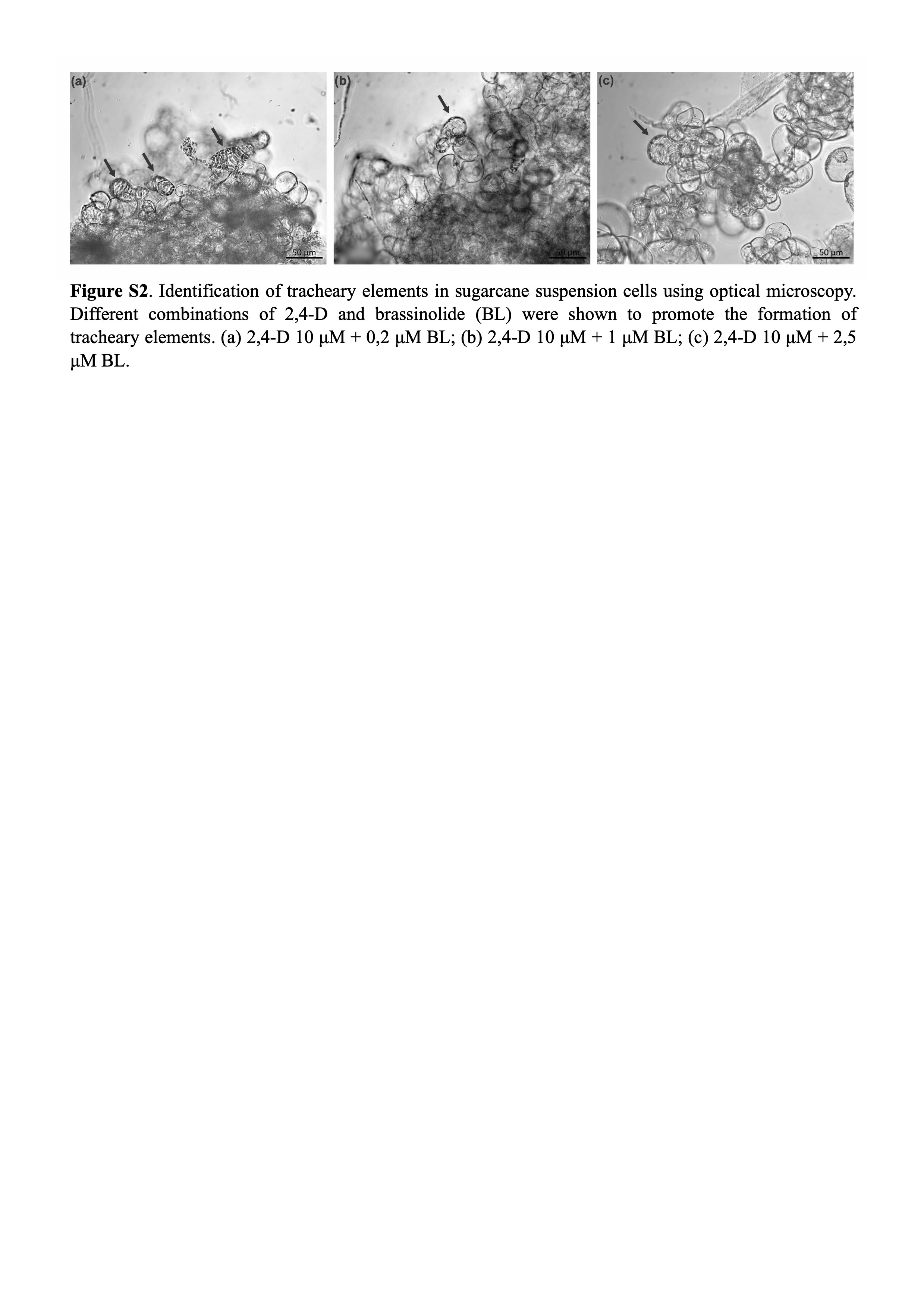

Supplement: Supplementary file 2 [file Image_2.JPEG]

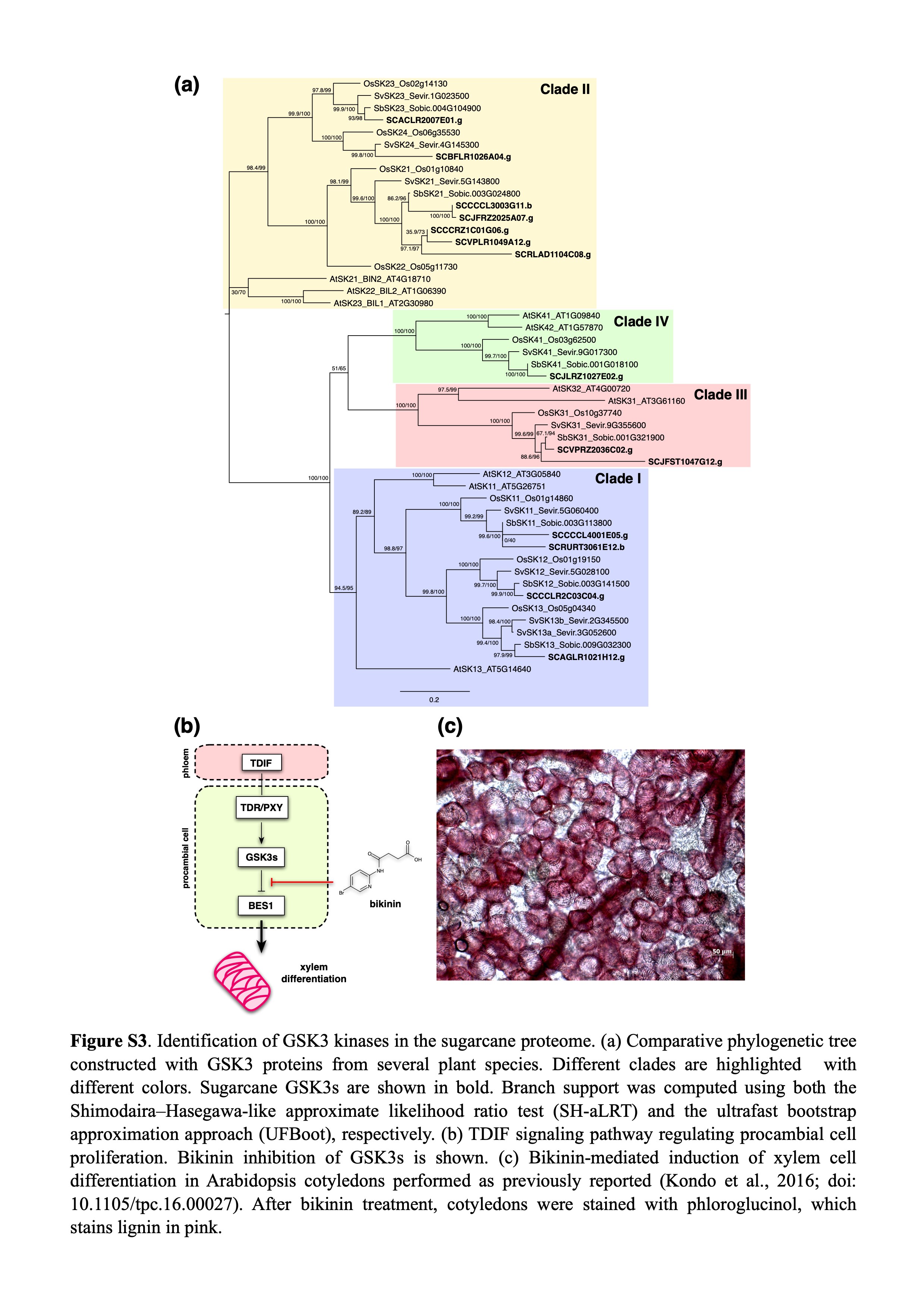

Supplement: Supplementary file 3 [file Image_3.JPEG]

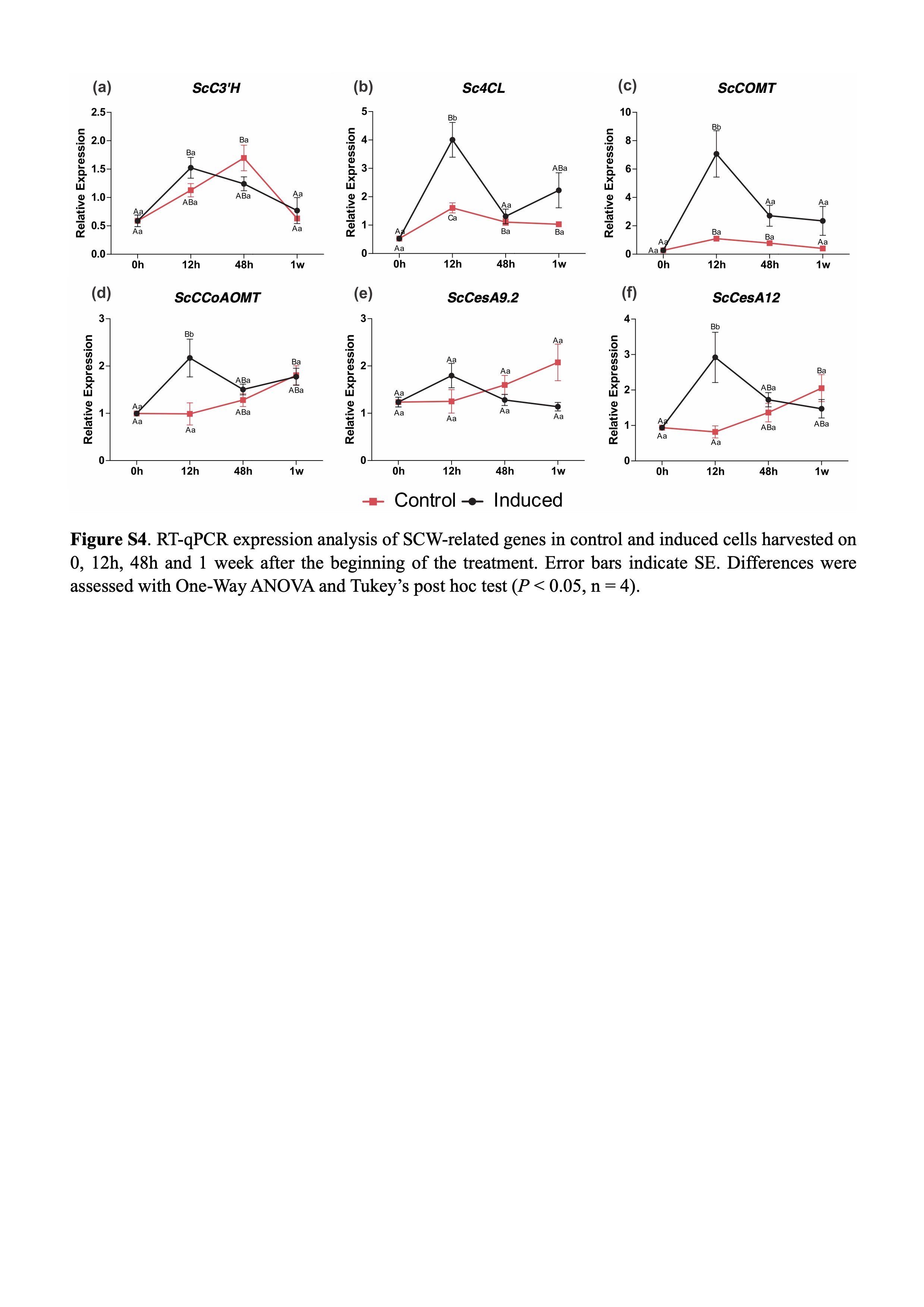

Supplement: Supplementary file 4 [file Image_4.JPEG]

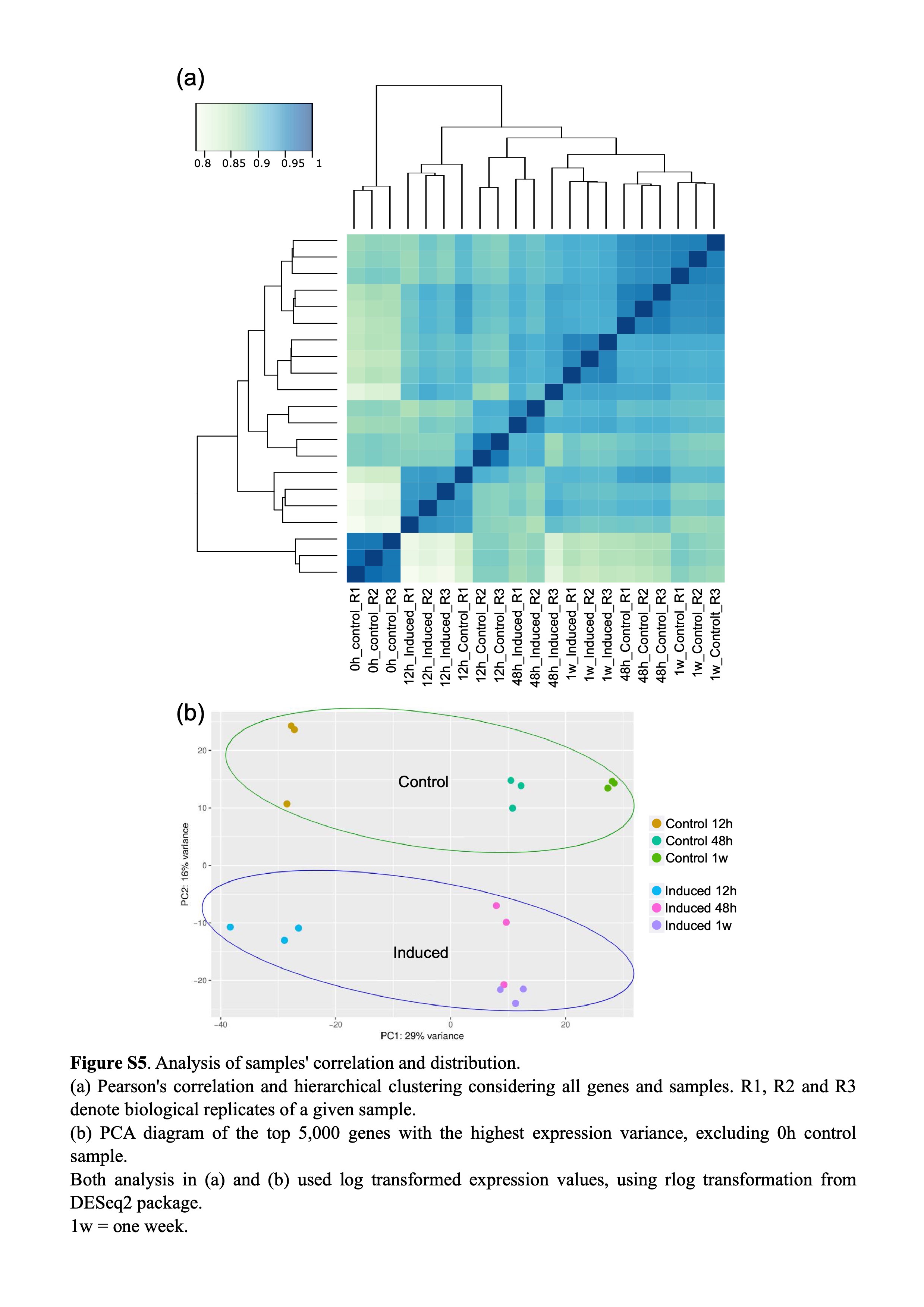

Supplement: Supplementary file 5 [file Image_5.JPEG]

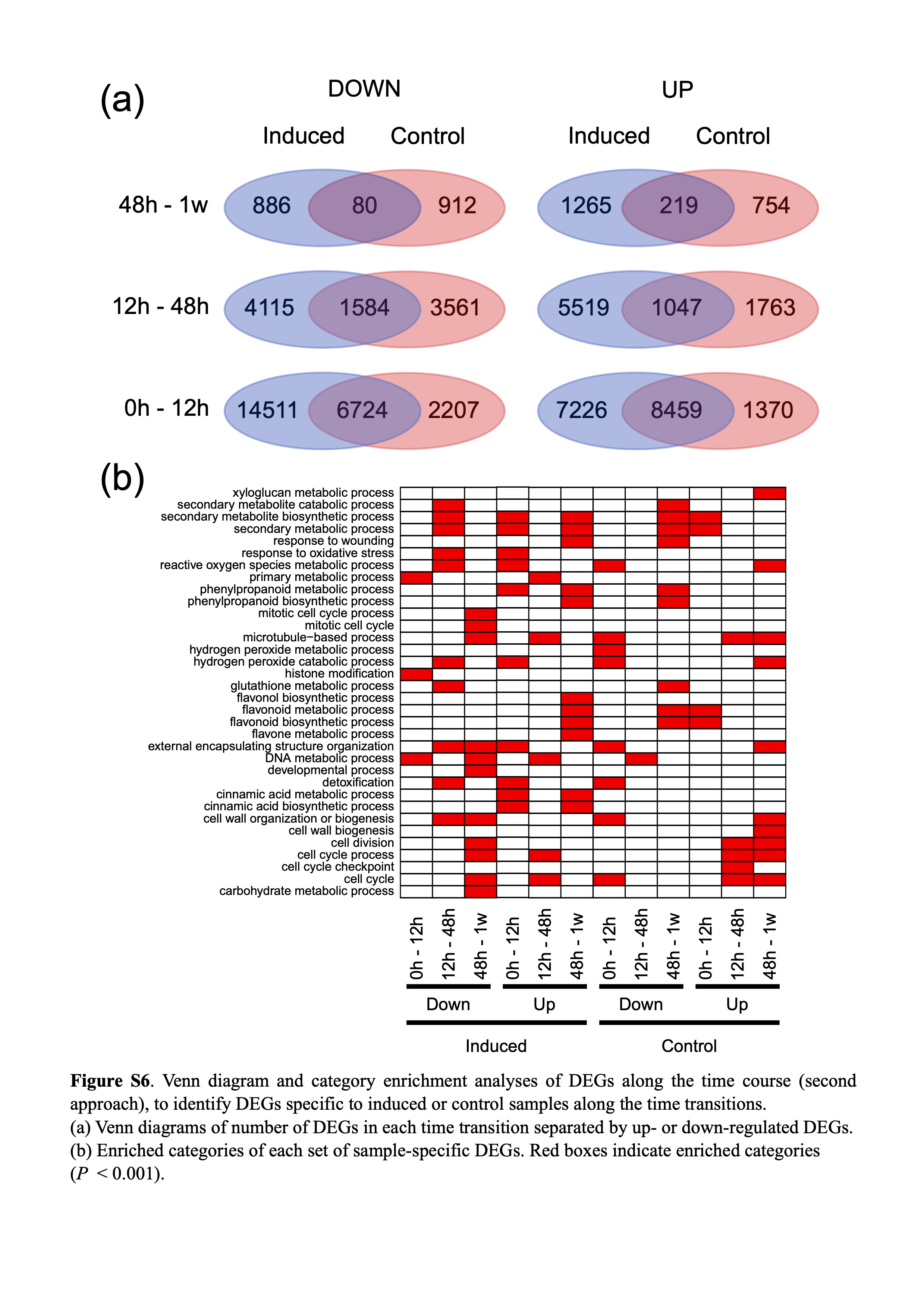

Supplement: Supplementary file 6 [file Image_6.JPEG]

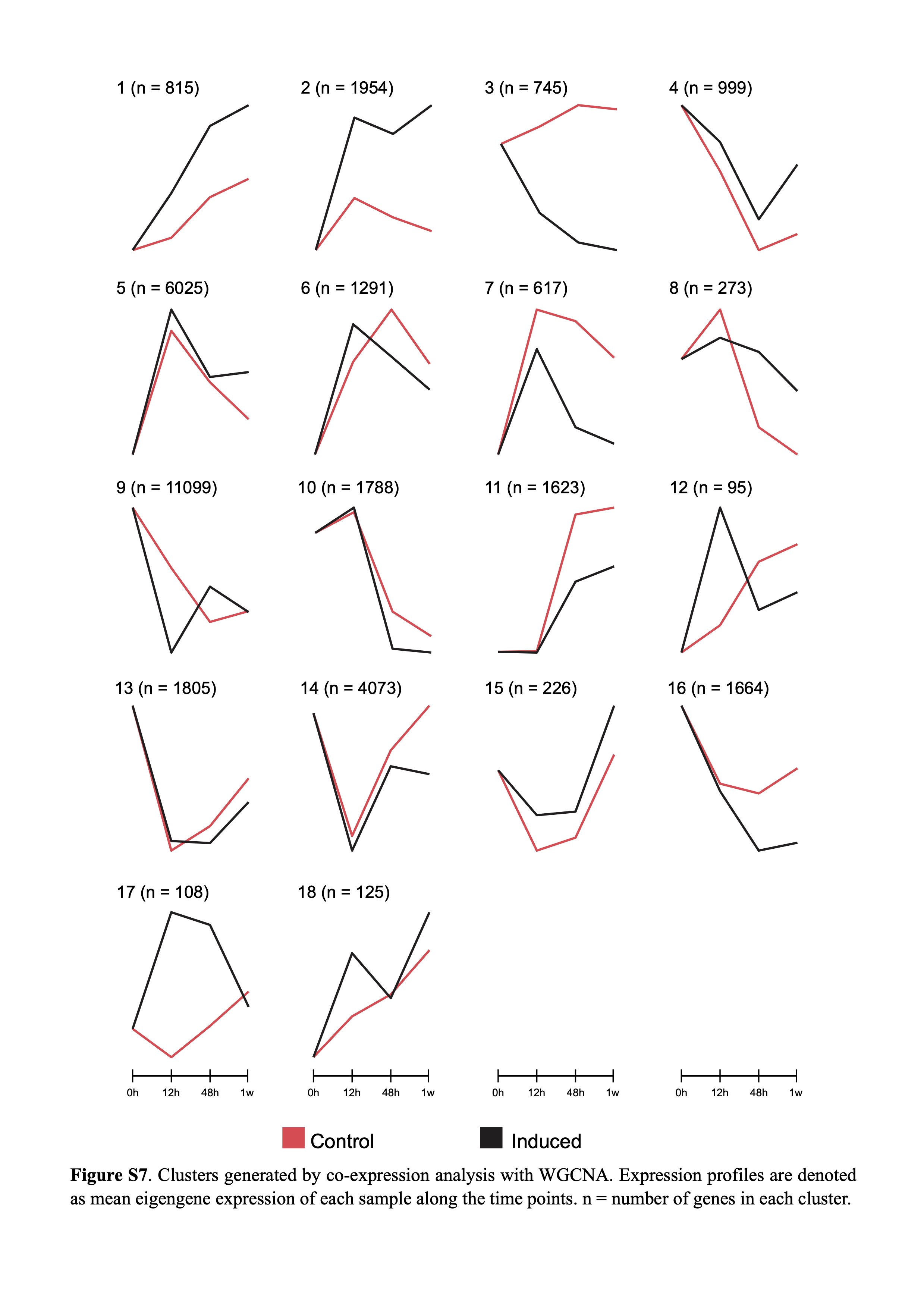

Supplement: Supplementary file 7 [file Image_7.JPEG]

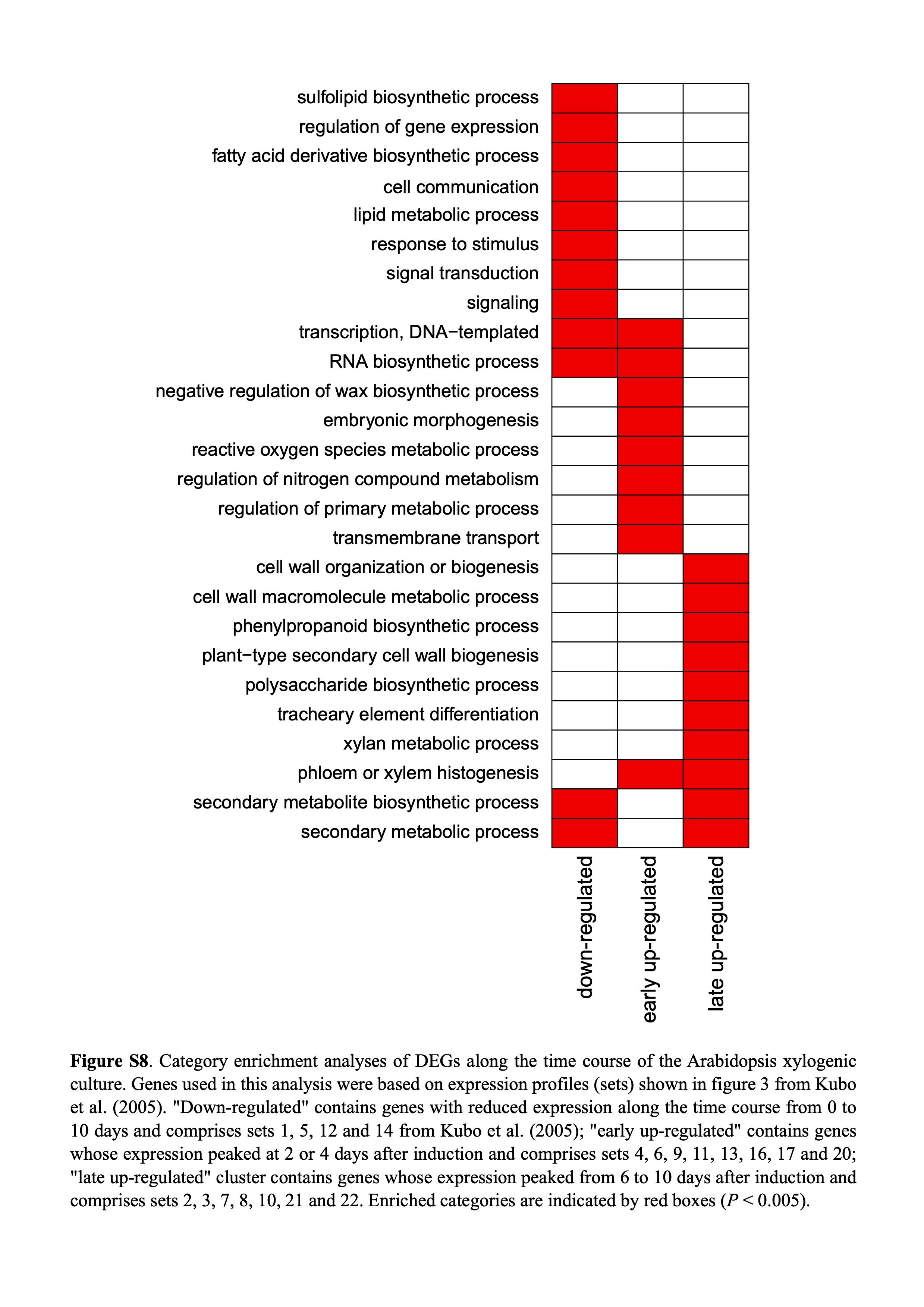

Supplement: Supplementary file 8 [file Image_8.JPEG]

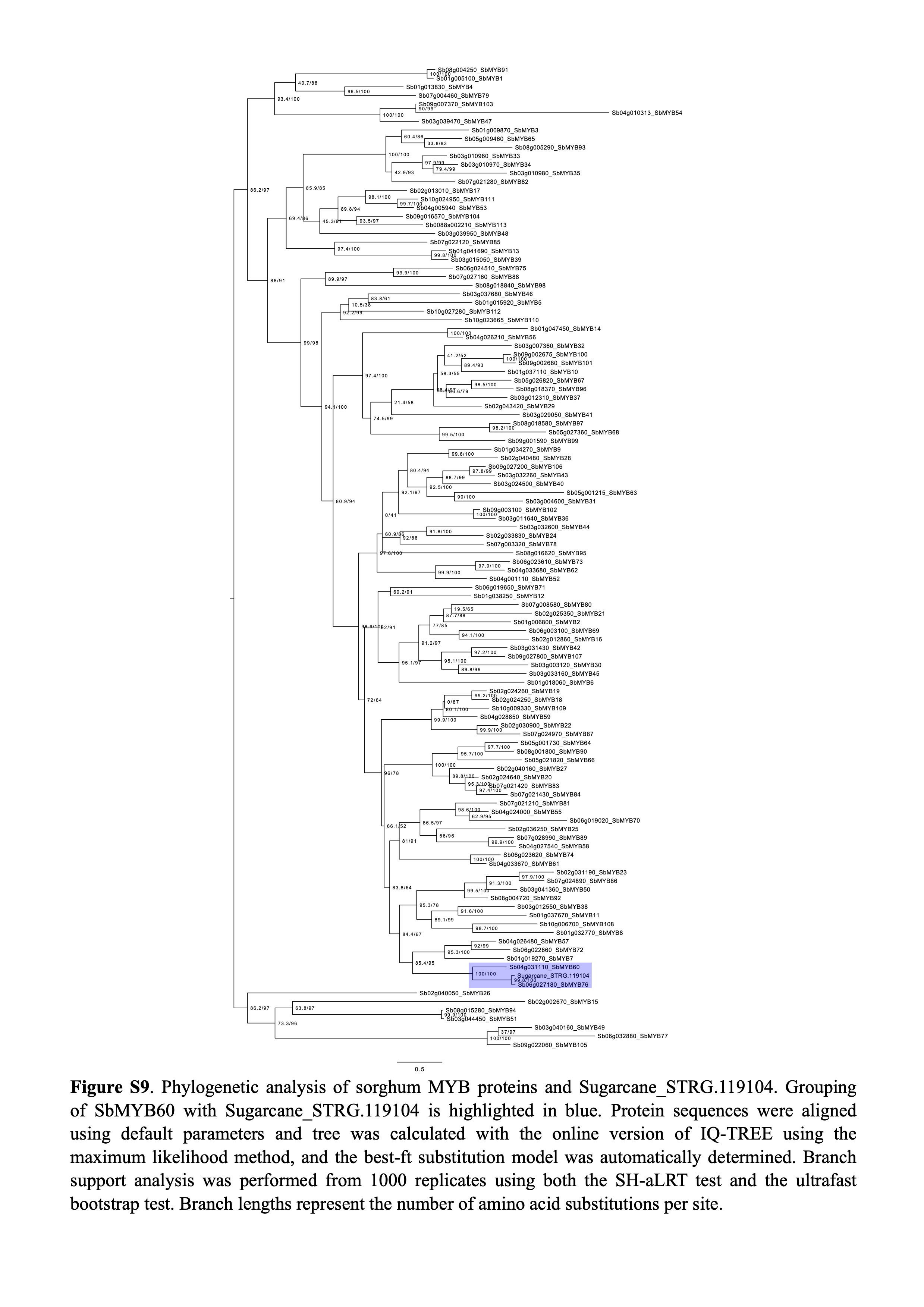

Supplement: Supplementary file 9 [file Image_9.JPEG]
