# Supplementary material for: Differentiation of Tracheary Elements in Sugarcane Suspension Cells Involves Changes in Secondary Wall Deposition and Extensive Transcriptional Reprogramming
Source: Front Plant Sci. 2020 Dec 18;11:617020. doi: 10.3389/fpls.2020.617020 (PMC7814504; doi:10.3389/fpls.2020.617020)
Supplement: Supplementary file 10 [file Table_1.DOCX]

**Table S1**. Assignments of the lignin correlation signals in the 2D HSQC spectra of sugarcane suspension cells. Signals were assigned by comparison with literature (del Río et al., 2012, 2015).

| **Label** | **δ_C_/δ_H_ (ppm)** | **Assignment** |
| --- | --- | --- |
|  |  |  |
| S_2,6_ | 103.8/6.69 | C_2_/H_2_ and C_6_/H_6_ in syringyl units (**S**) |
| G_2_ | 110.9/6.99 | C_2_/H_2_ in guaiacyl units (**G**) |
| FA_2_ | 110.9/7.31 | C_2_/H_2_ in ferulates (**FA**) |
| *p*CA_8_ and FA_8_ | 113.9/6.48 | C_8_/H_8_ in *p*-coumarates (***p*CA**) and ferulates (**FA**) |
| G_5_/_6_ | 114.9/6.72 | C_5_/H_5_ and C_6_/H_6_ in guaiacyl units (**G**) |
| FA_5_ | 115.3/6.77 | C_5_/H_5_ in ferulates (**FA**) |
| *p*CA_3,5_ | 115.5/6.77 | C_3_/H_3_ and C_5_/H_5_ in *p*-coumarates (***p*CA**) |
| G_6_ | 119.0/6.76 | C_6_/H_6_ in guaiacyl units (**G**) |
| FA_6_ | 123.1/7.11 | C_6_/H_6_ in ferulates (**FA**) |
| *p*CA_2,6_ | 130.1/7.45 | C_2_/H_2_ and C_6_/H_6_ in *p*-coumarates (***p*CA**) |
| *p*CA_7_ and FA_7_ | 145.2/7.56 | C_7_/H_7_ in *p*-coumarates (***p*CA**) and ferulates (**FA**) |
|  |  |  |
