# Supplementary material for: Differentiation of Tracheary Elements in Sugarcane Suspension Cells Involves Changes in Secondary Wall Deposition and Extensive Transcriptional Reprogramming
Source: Front Plant Sci. 2020 Dec 18;11:617020. doi: 10.3389/fpls.2020.617020 (PMC7814504; doi:10.3389/fpls.2020.617020)
Supplement: Supplementary file 11 [file Table_2.DOCX]

**Table S2.** Semiquantitative 2D-NMR analysis (relative abundances of lignin units and hydroxycinmamates, as per total lignin and carbohydrate units) of the sugarcane control and induced cells. Data for two biological replicates are shown.

|  | Control-1 | Control-2 | Treated-1 | Treated-2 |
| --- | --- | --- | --- | --- |
|  |  |  |  |  |
| total lignin units | 2.3 | 2.7 | 7.5 | 7.1 |
| guaiacyl lignin units (**G**) | 1.2 | 1.3 | 3.1 | 2.9 |
| syringyl lignin units (**S**) | 1.1 | 1.4 | 4.4 | 4.2 |
| **S**/**G** ratios | 0.92 | 1.08 | 1.42 | 1.45 |
| *p*-coumarates (***p*CA**) | 1.0 | 1.3 | 9.7 | 9.1 |
| ferulates (**FA**) | 9.8 | 10.1 | 7.8 | 7.2 |
| ***p*CA**/**FA** ratios | 0.11 | 0.13 | 1.24 | 1.27 |

^*^Sample composition represents the abundance of lignin units (**G**, **S**) and hydroxycinnamates (***p*CA**, **FA**) from integration of their respective signals, and carbohydrate units (xylose, glucose and 4-*O*-methyl-α-D-glucuronic acid) from integration of the anomeric carbon signals, and are referred to the total lignin (**G** + **S**) and carbohydrate units (lignin + carbohydrate units = 100). *p*-Hydroxyphenyl (**H**) units were not quantified due to overlapping with signals from proteins.
